# Chromosome-scale genome assembly for the duckweed *Spirodela intermedia*, integrating cytogenetic maps, PacBio and Oxford Nanopore libraries

**DOI:** 10.1038/s41598-020-75728-9

**Published:** 2020-11-05

**Authors:** Phuong T. N. Hoang, Anne Fiebig, Petr Novák, Jiří Macas, Hieu X. Cao, Anton Stepanenko, Guimin Chen, Nikolai Borisjuk, Uwe Scholz, Ingo Schubert

**Affiliations:** 1 Leibniz Institute of Plant Genetics and Crop Plant Research (IPK), 06466 Gatersleben, Stadt Seeland, Germany; 2 Biology Faculty, Dalat University, District 8, Dalat City, Lamdong Province Vietnam; 3 Biology Centre, Czech Academy of Sciences, Institute of Plant Molecular Biology, České Budějovice, 37005 Czech Republic; 4 Jiangsu Key Laboratory for Eco-Agricultural Biotechnology Around Hongze Lake, School of Life Sciences, Huaiyin Normal University, Huai’an, 223300 China; 5 Jiangsu Collaborative Innovation Centre of Regional Modern Agriculture and Environmental Protection, Huaiyin Normal University, Huai’an, 223300 China; 6 Present Address: Institute of Biology, Martin-Luther-University Halle-Wittenberg, 06120 Halle, Germany

**Keywords:** Evolution, Genetics

## Abstract

Duckweeds are small, free-floating, morphologically highly reduced organisms belonging to the monocot order Alismatales. They display the most rapid growth among flowering plants, vary ~ 14-fold in genome size and comprise five genera. Spirodela is the phylogenetically oldest genus with only two mainly asexually propagating species: *S. polyrhiza* (2n = 40; 160 Mbp/1C) and *S. intermedia* (2n = 36; 160 Mbp/1C). This study combined comparative cytogenetics and de novo genome assembly based on PacBio, Illumina and Oxford Nanopore (ON) reads to obtain the first genome reference for *S. intermedia* and to compare its genomic features with those of the sister species *S. polyrhiza*. Both species’ genomes revealed little more than 20,000 putative protein-coding genes, very low rDNA copy numbers and a low amount of repetitive sequences, mainly Ty3/gypsy retroelements. The detection of a few new small chromosome rearrangements between both *Spirodela* species refined the karyotype and the chromosomal sequence assignment for *S. intermedia.*

## Introduction

Duckweeds are the smallest and fastest-growing flowering plants, and are considered as potential aquatic crops, serving for feed, food, fuel and waste water remediation^1–13^. They comprise 36 largely asexually propagating species within the 5 genera Spirodela, Landoltia, Lemna, Wolffiella and Wolffia^14–16^ With decreasing phylogenetic age duckweed frond sizes decrease from 1.5 cm to less than 1 mm in diameter accompanied by a successive reduction or loss of roots and a nearly 14-fold genome size variation (from 160 to 2203 Mbp)^17–20^. These features make duckweeds an interesting subject for genome and karyotype evolution studies. So far, no correlation between genome size, chromosome number as well as ribosomal DNA loci was recorded from eleven species representative for five duckweed genera^18^.

The genus Spirodela harbors only two species of similar genome size (160 Mbp), *S. polyrhiza* and S*. intermedia*. Due to its basal ancestral phylogenetic position, its industrial potential and its small genome, the Greater Duckweed *S. polyrhiza* was chosen as the first duckweed for whole genome sequencing^21^. By integrating different approaches: cytogenetics, optical mapping (BioNano technique), Hi-C chromatin conformation study, 454, Illumina and Oxford Nanopore sequencing platforms, a high-confidence genome map for *S. polyrhiza* was established^22,24^ that corrected the errors of previous genome maps^21,23–25^. This high-quality genome map provides a source for advanced genomic research regarding repetitive sequences and protein-coding genes, their chromosomal location and evolutionary history in other duckweeds. Moreover, whole-genome duplication (WGD) events and chromosomal rearrangement between duckweed species can potentially be uncovered using the *S. polyrhiza* genome as a reference. Between the chromosomes of seven cytogenetically investigated *S. polyrhiza* clones so far no BAC-sized structural rearrangements were found^24^. In addition, population genomics studies suggested a considerably low genetic diversity between world-wide distributed *S. polyrhiza* clones^26,27^.

Sequence assignment to distinct chromosomes based on cross-hybridization of genomic sequences between related species represents a novel cytogenomic approach*.* Such an approach is particularly important for vegetatively propagating species*,* for which obtaining a genetic map to validate sequence assembly is difficult. However, in such cases at least one reference genome for validating bioinformatic assembly efforts is required, and a considerable number of cytogenetic anchor points should provide a reliable support for sequence data integration as previously exemplified for *Amborella trichopoda*^28^ and for *S. polyrhiza*^23^. These prerequisites are given for *S. intermedia.* In our previous study, chromosome homeology and rearrangements between *S. polyrhiza* (2n = 40) and *S. intermedia* (2n = 36) were investigated by cross-FISH with 93 anchor BACs of *S. polyrhiza*^29^. Thus, a high-confidence genome map of *S. polyrhiza* as a reference, and a cytogenetic map of *S. intermedia* are available to support genomic sequence assembly from reads generated by next-generation sequencing (NGS) platforms.

In this study, the cytogenetic maps of two *S. intermedia* clones (8410 and 7747) served as a frame for whole genome assembly of the vegetatively propagating *S. intermedia.* The same chromosomal rearrangements distinguishing clone 8410 from *S. polyrhiza* were also found for clone 7747 applying 93 anchored BACs. By integrating the cytogenetic maps and genome assembly from PacBio reads for clone 7747 and Illumina/ON reads for clone 8410, we generated a robust, chromosome-scaled genome map, apparently identical for both *S. intermedia* clones and revealed additionally further small evolutionary rearrangements between the two Spirodela species.

## Results

### The cytogenetic map for *S. intermedia* clone 7747

**Figure 1:**
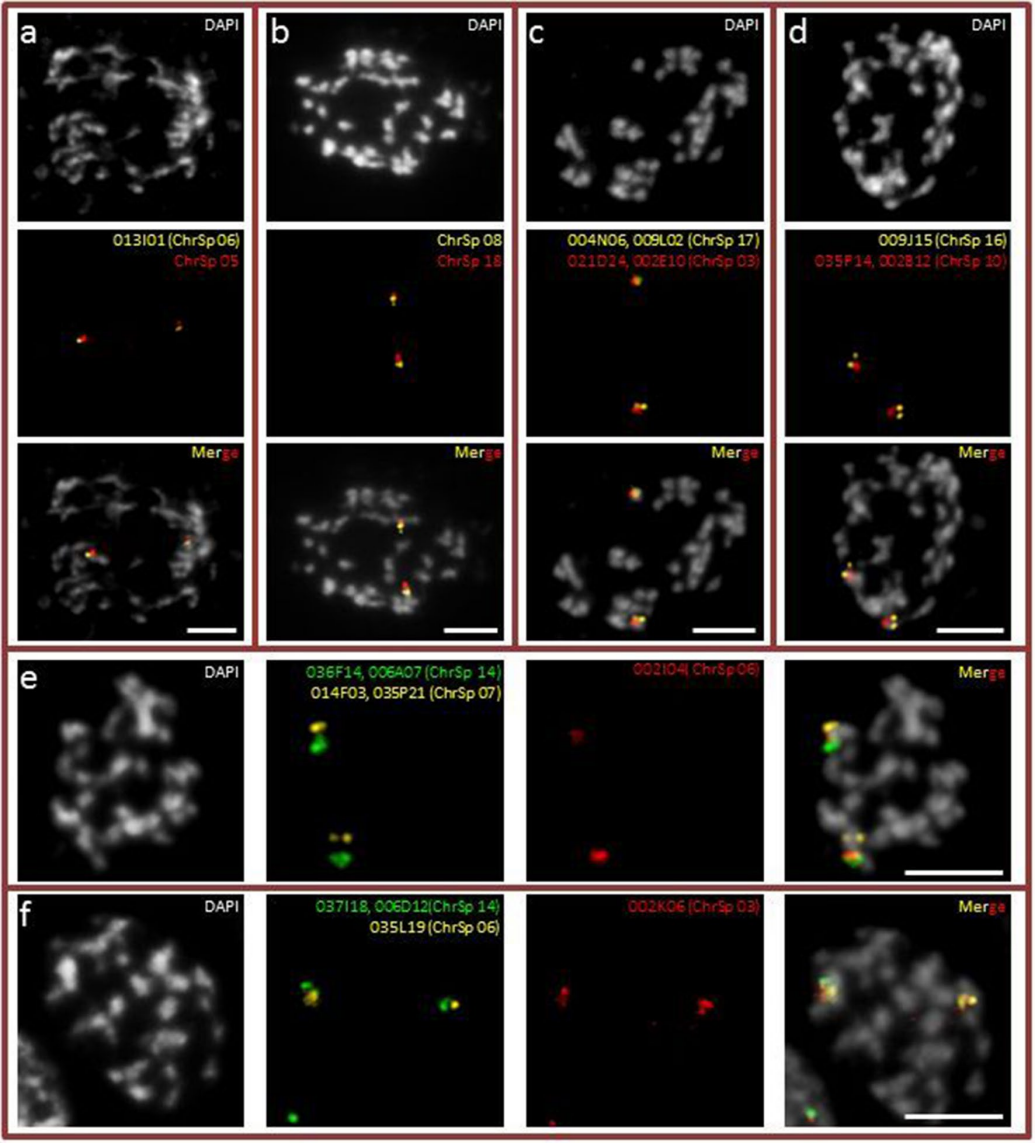
Six linkages due to chromosome rearrangements between *S. polyrhiza and S. intermedia* (clone 8410) are also present in the sequenced clone 7747. (**a**) ChrSp05–ChrSp06 = ChrSi06; (**b**) ChrSp08–ChrSp18 = ChrSi09; (**c**) ChrSp03–ChrSp17 = ChrSi04; (**d**) ChrSp10–ChrSp16 = ChrSi11; (**e**) ChrSp06–ChrSp07–ChrSp14 = ChrSi07; (**f**) ChrSp03–ChrSp06–ChrSp14 = ChrSi03. See also Fig. 3. Scale bars = 5 µm.

Previously we established a cytogenetic map for *S. intermedia* clone 8410 using 93 BACs anchored in the *S. polyrhiza* genome^29^. Now we hybridized diagnostic BACs to the chromosomes of the *S. intermedia* clone 7747 in order to test whether chromosomal differences occurred between the karyotypes of these two *S. intermedia* clones, because different chromosome numbers (2n = 36 versus 2n = 20) have been reported for *S. intermedia* clone 7747^30,31^. Our current chromosome counting found no chromosome number difference between clones 7747 and 8410. For both clones 2n = 36 were counted^18^ (Fig. S1). In order to test whether structural chromosomal rearrangements occurred between these *S. intermedia* clones, we applied cross-FISH with suitable combinations of 93 *S. polyrhiza* BACs, as described in Hoang & Schubert, 2017^29^, on mitotic spreads of *S. intermedia* clone 7747. We used the same chromosome designation as in Hoang & Schubert, 2017^29^: ChrSp for chromosomes of *S. polyrhiza* and ChrSi for chromosomes of *S. intermedia*. No chromosomal rearrangements between these two *S. intermedia* clones were detected. Six linkages in *S. intermedia* that differed from the *S. polyrhiza* karyotype [ChrSp03–ChrSp06–ChrSp14]; [ChrSp05–ChrSp06]; [ChrSp06–ChrSp07–ChrSp14]; [ChrSp03–ChrSp17], [ChrSp10–ChrSp16] and [ChrSp08–ChrSp18] previously reported for clone 8410^29^, were found also for clone 7747 (Fig. 1). The similarity between the cytogenetic maps of *S. intermedia* clones 7747 and 8410 enabled merging of genome assembly from PacBio reads for clone 7747 and of Illumina and ON reads for clone 8410, and yielded an apparently identical genome map for both *S. intermedia* clones*.*

### Genome assembly for *S. intermedia* clone 7747 based on the library of PacBio sequence reads

Two rounds of PacBio-sequencing of a 20 kb library of genomic DNA of *S. intermedia* clone 7747 resulted in 149 Gbp of raw read data. After an initial filtering for potential bacterial contamination, reads of at least 500 nucleotides were assembled using the Canu pipeline v. 1.5^32^.

A total of 1,305,064 reads were assembled into 1172 sequence contigs of 147,613,042 nucleotides, corresponding 91.7% of the estimated genome size*.* All contigs of this draft assembly are covered in median 37.5-fold by raw reads. In a first round of scaffolding, the two genomes of the sister species *S. polyrhiza* (from clones 9505 and 7498)^23,25^ were used as references to order contigs as described^33^. The resulting scaffolds (N50 = 1,7 Mbp) were super-scaffolded by SSPACE-Longread v.1–1^34^ and assigned to the 18 chromosomes of *S. intermedia,* using 93 *S. polyrhiza* BACs as landmarks which were cross-hybridized to the *S. intermedia* chromosomes of clone 8410^29^. In addition to confirmation of the same linkage relationship in clone 7747 (Fig. 1), new cytogenetic probes using BACs from the genomic regions of interest were designed for FISH experiments to approve localization of the contigs within the pseudomolecules, to resolve mis-assemblies and/or to confirm new linkages (see below). Furthermore, contiguity of the assembly was confirmed by corrected ON reads of *S. intermedia* clone 8410 using minimap2 v2.16^35^ (see below).

**Figure 2:**
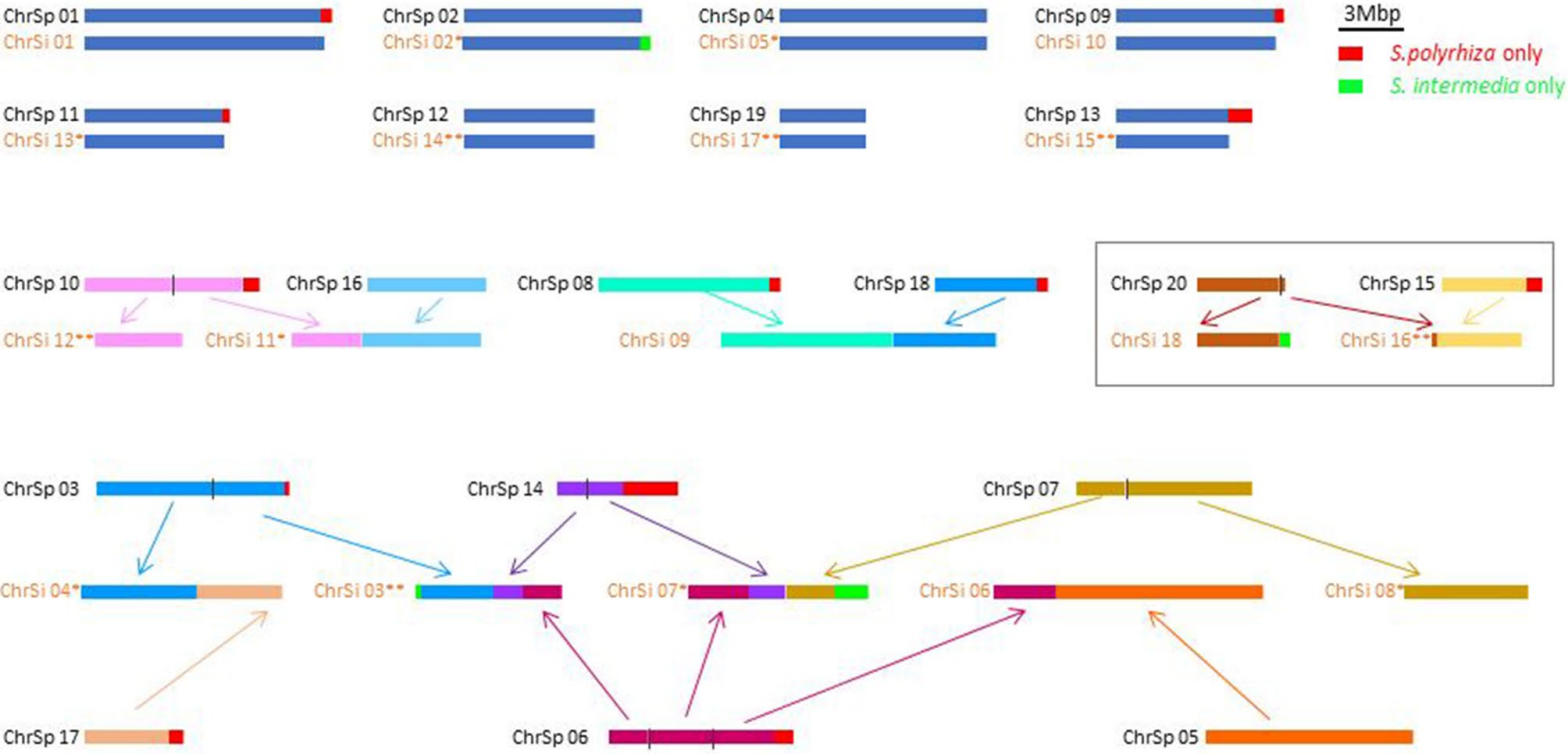
Rearrangements between *S. polyrhiza* (n = 20) and *S. intermedia* (n = 18), confirmed for chromosomes of clones 8410 and 7747. Enframed: newly found rearrangement. Red boxes: present only in *S. polyrhiza*; green boxes: sequences present only in *S. intermedia.* Scale bar = 3 Mbp (based on PacBio assembly for clone 7747). Enumeration is as in Hoang & Schubert (2017)^29^ and in Table S3 (Short sequences present in S. p., but not chromosomally assigned in S. i. correspond to “SiUn” and are not considered). 13 ChrSi of the 7747 assembly show telomeric sequences at one (*) or both ends (**), while all pseudomolecules of the 8410 assembly show them at both ends.

After reiterative rounds of manual curation and validation by FISH, in the final genome assembly, 18 scaffolds (N50 = 8.3 Mbp) of in total 131.4 Mbp (82.2% of the estimated genome size) could be assigned to the 18 chromosomes (Table 1). Six of them show telomeric sequences at both ends and seven at least on one end (see Fig. 2). Most of the shorter and/or repetitive sequences (16.2 Mbp, corresponding 10.1% of the estimated genome size) (N50 = 27.1 Kbp) could not yet be assigned and were considered as additional pseudomolecule “SiUn”.

**Table 1:** Assembly statistics of *S. intermedia* clones.

Sequencing technique	PacBio (clone 7747)	ON/Illumina (clone 8410)
**Input data**		
Read coverage^a^	37.6×	191×
Chromosome number	18	
Genome physical size^b^	~ 160 Mbp	
**Assembly statistics and gene prediction**		
Assembly length (Mbp)	147.6	136.6
Number of pseudomolecules	18	
Number of contigs	584	86
Number of scaffolds	420	70
Number of assigned scaffolds	18 pseudomolecules (featuring 63 contigs) ~ 131.4 Mbp total length	18 pseudomolecules (featuring 34 contigs) ~ 134 Mbp total length
Largest scaffold length (Mbp)	12.5 (Si09)	13.4 (Si09)
N50 scaffold length (Mbp)	8.3	9.25
G + C content (%)	41.6	42.0
Number of predicted gene models	22,245	21,594
**Completeness of gene prediction (BUSCO)**^**c**^		
Complete genes (C)	1097 (79.8%)	1280 (93.1%)
Complete and single-copy (S)	1085 (78.9%)	1266 (92.1%)
Complete and duplicated (D)	12 (0.9%)	14 (1.0%)
Fragmented genes (F)	131 (9.5%)	41 (3.0%)
Missing genes (M)	147 (10.7%)	54 (3.9%)
Total number of BUSCO genes used	1375	

^a^Based on genome size measurements by FCM.

^b^Measured by FCM.

^c^Reference database odb10.

### Genome assembly for *S. intermedia* clone 8410 based on Illumina and Oxford Nanopore reads

**Figure 3:**
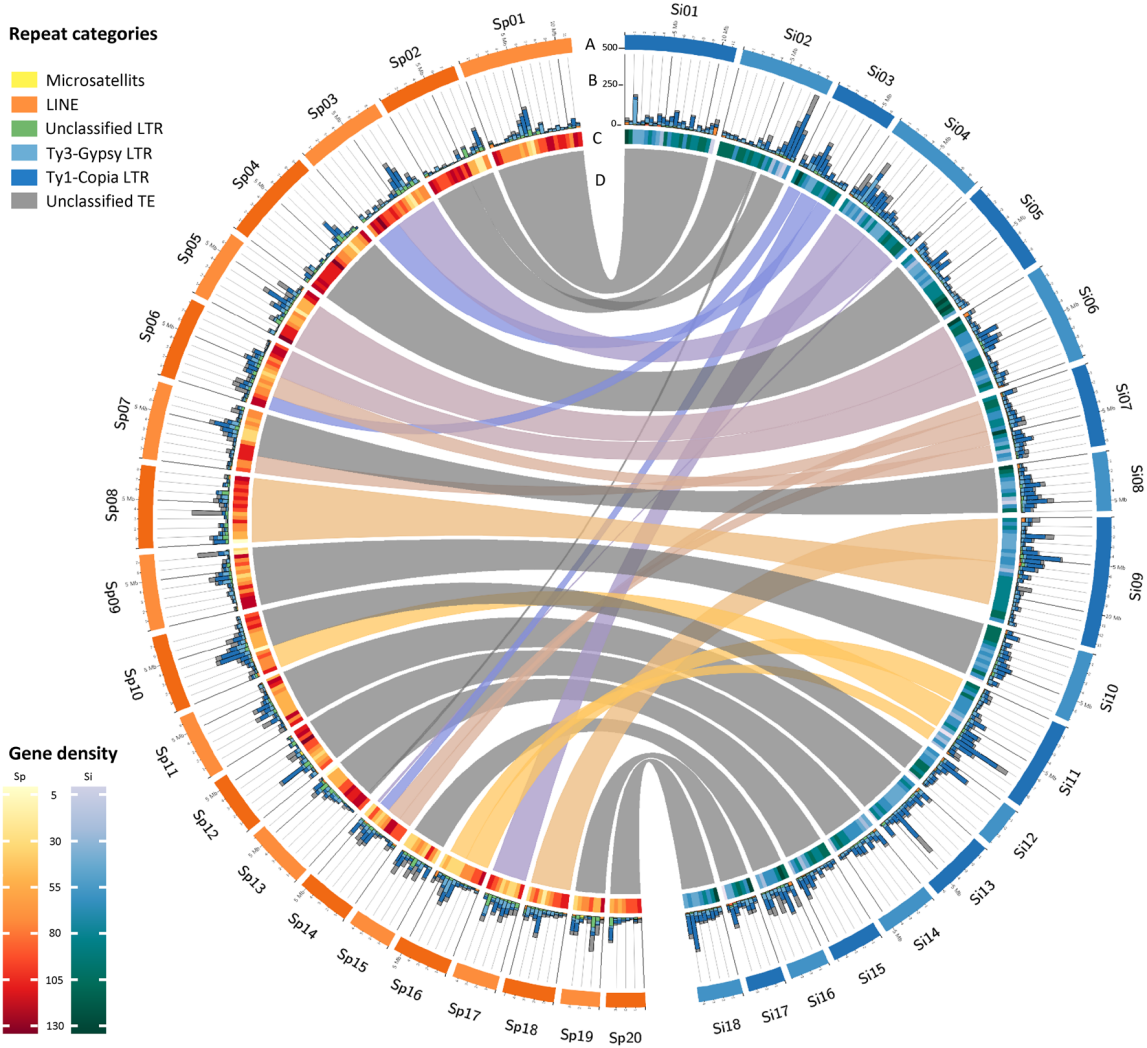
Circos plot of genomes of *S. polyrhiza* 9505 (orange) and *S. intermedia* 8410 (blue). (**A**) tracks representing the size of the pseudomolecules with a corresponding scale in 1 Mbp steps, with highlights every 5 Mbps, (**B**) total length of repeat features (in kbps) (**C**) gene density and (**D**) pairwise sequence synteny. The synteny link between gi|13 and Si02 is based on an error in the ON assembly for clone 9509 (see Fig. 5). The region in question actually belongs to gi|2 as does the remaining part of Si02. Gene and repeat density are plotted in 0.5 Mbps bins. Data for *S. polyrhiza* 9505 are from Michael et al. 2017^25^.

The ON/Illumina-derived scaffolds for clone 8410 were created using the MaSuRCA assembler and filtered using minimap2 to remove duplicated sequences derived from heterozygous regions. To form pseudomolecules, all 70 remaining scaffolds were corrected and ordered by manual curation using the assembly of clone 7747 as a reference. Finally, all 18 pseudomolecules ended at both sites with telomeric sequences. Merging of both assemblies revealed further small rearrangements between the karyotypes of *S. polyrhiza* and *S. intermedia* (see below and Figs. 2 and 3). The quality of both *S. intermedia* genome assemblies was assessed by the BUSCO program v. 3.1.0 ^36,37^ with Embryophyta dataset 10 including 1375 genes (mostly from land plants). The hybrid assembly of ON/Illumina reads revealed 96.1% of the 1375 searched genes (Table 1).

### Gene prediction

Based on similarity to nine aquatic and non-aquatic angiosperm reference genomes, including two duckweed species, *S. polyrhiza* 7498 v3.1^25^ and *Lemna minor* 5500^38^, gene model prediction via Gene Model Mapper—GeMoMa^39^ suggested in total 22,245 (Pacbio, clone 7747) or 21,594 (ON/Illumina, clone 8410) protein/RNA-coding genes, some more than predicted for *S. polyrhiza*^25^ (Table S1).

A total number of 16,162 genes of clone 7747, 16,493 of clone 8410 and 11,327 of *S. polyrhiza* clone 9509 are coinciding with eggNOGs (Non-supervised Orthologous Groups). Comparing the proportion of eggNOG functional categories between the genomes of the two *Spirodela* species, the differences were < 1%. Only the category ‘Energy production and conversion’, is overrepresented in *S. intermedia* clone 7747 (4.2 versus 3.1% in clone 8410 and 2.6% in *S. polyrhiza*) and the category ‘Replication, recombination and repair’ in clone 8410 (5.3% versus 3.2% in clone 7747 and 2.3% in *S. polyrhiza*) (Fig. S2, Table S2).

### New linkages in *S. intermedia* as revealed by genome assembly and FISH

**Figure 4:**
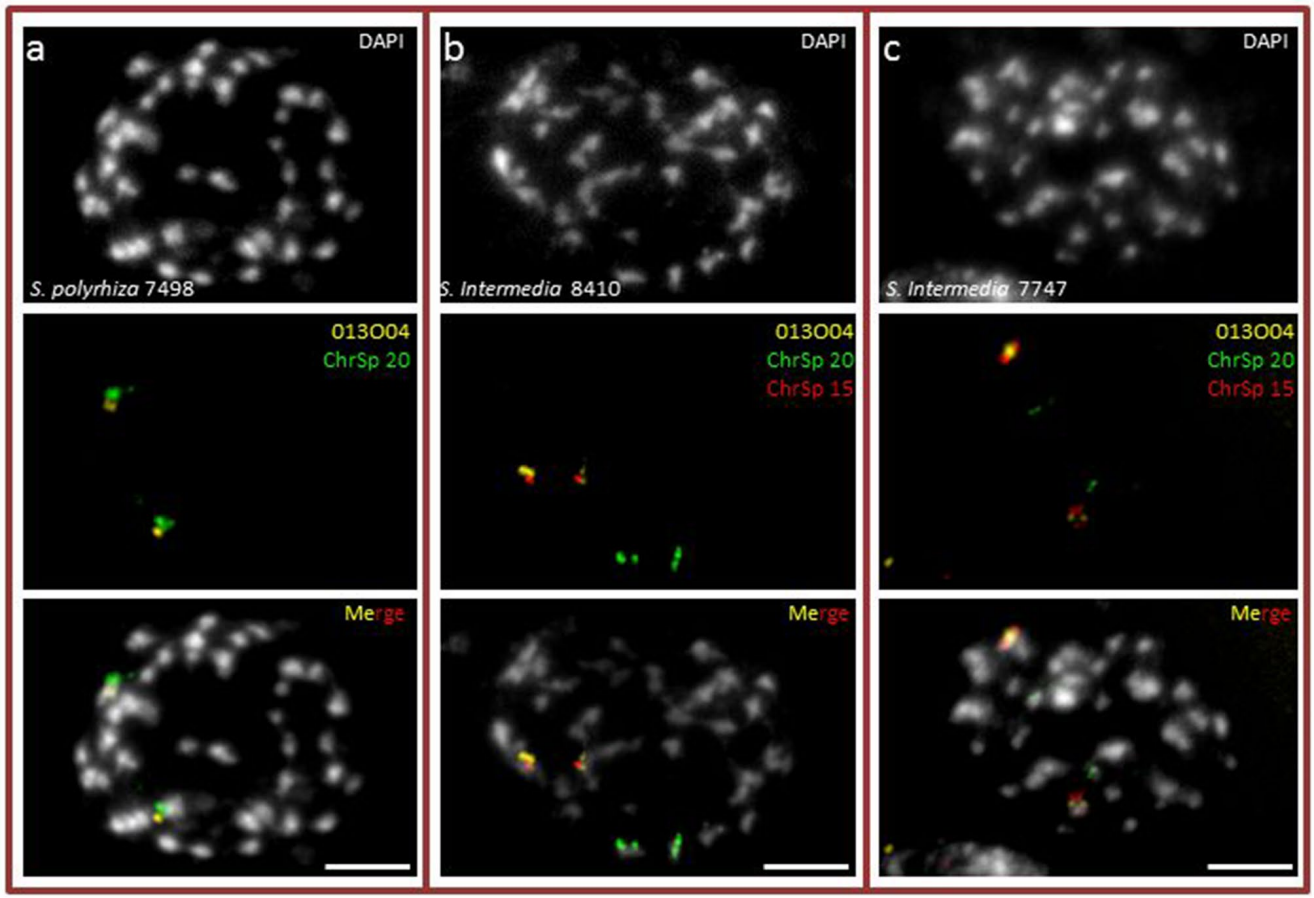
New rearrangement between ChrSp 20 and ChrSp 15 in *S. intermedia* (**a**) The newly tested BAC 013O04 (yellow) belongs to ChrSp 20 (green) in *S. polyrhiza*; (**b**,**c**) BAC 013O04 was translocated to ChrSp 15 (red) forming ChrSi 16 in *S. intermedia* clones 8410 and 7747. Scale bars = 5 µm.

The PacBio assembly for clone 7747 indicated a new rearrangement involving ChrSp20. This chromosome did not form the entire ChrSi18 as reported^29^, instead it is split into two parts, the largest part corresponding to ChrSi18 and two rather small regions (between ChrSp20 3.72–3.78 and 3.80–3.96 Mbp) transferred to ChrSi16 (42,000–175,000 bp) (Table S3). Therefore, the previously not tested BAC 013O04 belonging ChrSp20 was selected for mcFISH experiments. The presence of this BAC sequence in *S. polyrhiza* ChrSp20 was confirmed by FISH (Fig. 4a). FISH results on *S. intermedia* chromosomes (clone 8410: Fig. 4b, and clone 7747: Fig. 4c) showed that BAC 013O04 (yellow) labeled the same chromosome (ChrSi16) as the BACs belonging to ChrSp15 (red), while the remaining part of ChrSp20 (green) labeled another chromosome pair (ChrSi18). This result confirmed that ChrSp20 became split and a very small part became translocated to ChrSp15, forming ChrSi16 (Fig. 2), as inferred by PacBio assembly. Because only 76 kbp of the BAC 013O04 sequence appeared in the ON/Illumina assembly of ChrSi16, this link is not visible in Fig. 3 where the entire chromosome ChrSp20 is represented by the *S. intermedia* chromosome ChrSi18, based on the assembly results for clone 8410.

**Figure 5:**
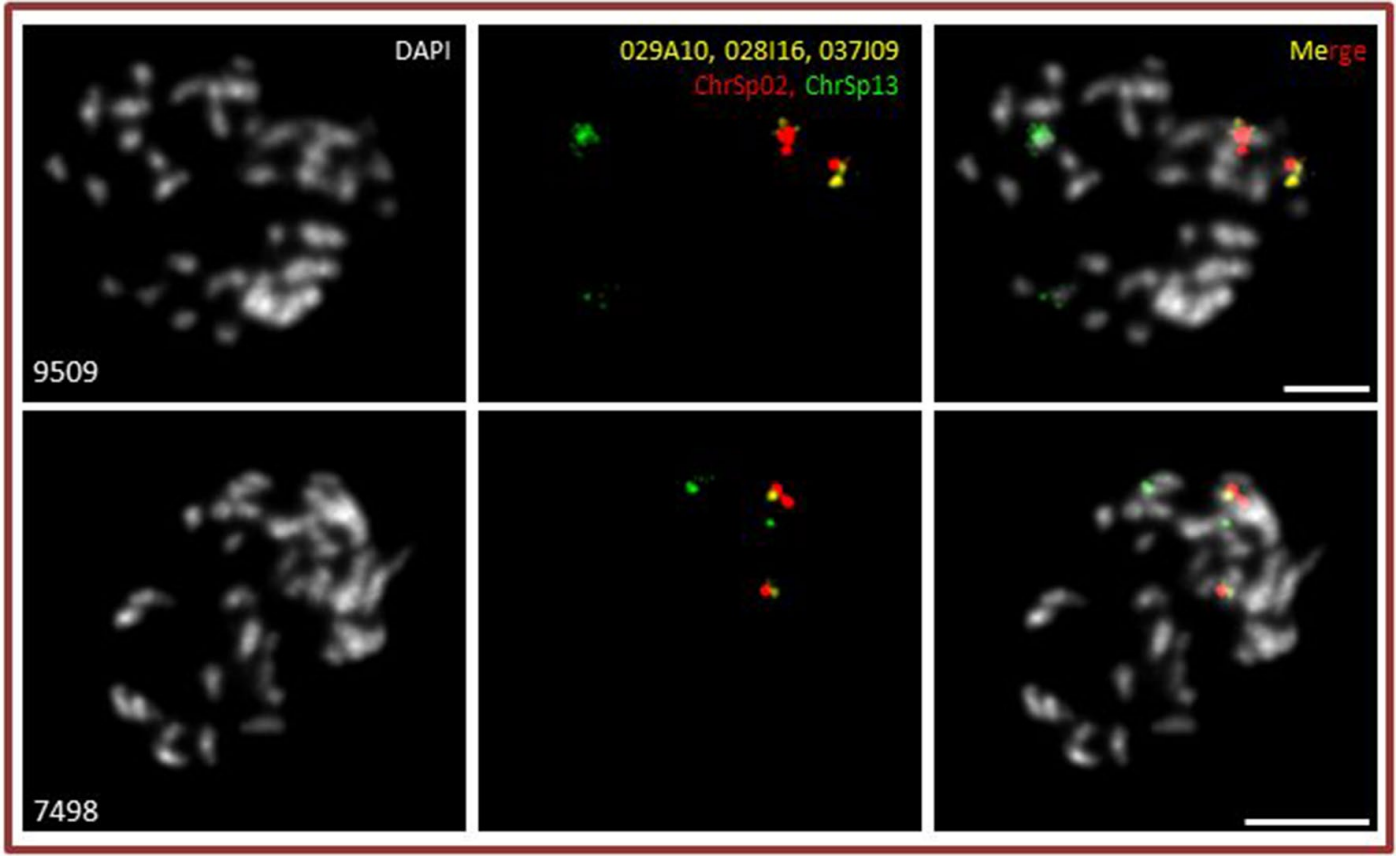
Evidence for ON mis-assembly of chromosome 13 of *S. polyrhiza* 9509. The newly tested BACs 029A10, 028I16 and 037J09 (yellow) belong to ChrSp 02 (red) of *S. polyrhiza* clone 9509 (upper panel) and clone 7498 (lower panel), not to ChrSp13. Scale bars = 5 µm.

Furthermore, a piece of ChrSp13 (0.46–0.68 Mpbs), according to the ON assembly for ChrSp13 of *S. polyrhiza* clone 9509, became integrated into ChrSi02 (5.43–5.66 Mbps) as suggested by the assemblies for both *S. intermedia* clones (for clone 8410 see Fig. 3). However, three BACs from this region (029A10, 028I16 and 037J09, together 649,507 bp) used as FISH probe, labeled ChrSp02 of *S. polyrhiza* clones 7498 and 9509 (Fig. 5) and appear on ChrSp02 also in the Bionano map (CP019095.1) of *S. polyrhiza* clone 9509^25^ as well as in pseudomolecule 1 (corresponding ChrSp02) of *S. polyrhiza* clone 7498^23^ (Table S4). This uncovers a hitherto overlooked error in the ON assembly for *S. polyrhiza* clone 9509^24^. Another new small region, for which no BAC is available, became transferred from ChrSp14 (4.9–5.4 Mbps) to ChrSi04 (9.5–9.9 Mbps) and appeared in both assemblies (for clone 8410 see Fig. 3).

Although only one BAC from ChrSp16 was tested previously^29^, the assemblies from PacBio and ON reads suggests that the entire ChrSp16 is included in *S. intermedia* ChrSi11 together with a part of ChrSp10 (Figs. 2, 3).

### Types, abundance and distribution of repetitive elements

Characterization of *S. intermedia* repetitive sequences was first performed by analyzing unassembled Illumina reads from the clone 8410 using the RepeatExplorer pipeline^40^. This analysis served as a control for repeat quantification in the assembled pseudomolecules that may be biased due to the exclusion of satellite DNA or other repeats that are hard to assemble. In addition, the RepeatExplorer output was used to compile a reference database of *S. intermedia* repeats that was used to annotate the genome assemblies.

The RepeatExplorer analysis of 2.4 million paired-end Illumina reads (2.25 × genome coverage) revealed relatively small proportions of highly and moderately repeated sequences in the *S. intermedia* genome (Table 2). The repeats accounted for 20% of the genome, with LTR-retrotransposons representing the most abundant repeat class (14.1% of the genome, with Ty3/gypsy to Ty1/copia ratio of 2.17). Other repeats including LINEs, rDNA and satellite DNA made up only minor genome proportions.

**Table 2:** Repeat proportions [%] estimated for unassembled sequence reads and genome assemblies of *S. intermedia* clones 8410 and 7747.

Repeat	8410	8410	7747
	Illumina reads	Assembly	Assembly
**Ty3/gypsy**			
Athila	7.44	7.23	8.57
CRM	1.09	1.02	1.13
Reina	0.36	0.32	0.30
Galadriel	0.05	0.04	0.05
Tekay	0.02	0.00	0.00
**Ty1/copia**			
Ale	1.87	1.78	1.79
Ivana	0.94	0.91	0.97
Tork	0.69	0.71	0.70
Ikeros	0.40	0.37	0.39
Unclassified	0.23	0.21	0.27
LTR unclass	1.01	0.93	1.08
LINE	0.37	0.42	0.49
Satellite	0.17	0.06	0.09
Microsat. (GA)n	0.67	1.02	0.98
rDNA	0.47	0.34	0.06
Unclassified	4.21	3.11	3.35
Total	20.00	18.49	20.22
RepeatScout	n.a	23.11	25.58

Repeat annotations of the genome assemblies using the RepeatExplorer reference database resulted in 18.5% and 20.2% of the 8410 and 7747 sequences marked as repetitive, respectively. These proportions correspond to the estimate obtained for unassembled reads (20.0%), suggesting that repeats were not significantly depleted in the final assemblies. Inspection of individual repeat categories revealed that partial depletion resulting in smaller proportions in the assembled genomes occurred for satellite repeats in both clones and for rDNA in the clone 7747 (Table 2). To obtain an alternative estimate of repeat proportions, we also analyzed both assemblies using RepeatScout^41^ that performs *de-novo* identification of repetitive elements based on the high frequency k-mers. This program estimated total repeat proportions of 23.1% (8410) and 25.6% (7747), most likely due to its better sensitivity for low-copy repeats (Table 2).

An interesting observation was the relatively high abundance of simple-sequence repeats, especially the microsatellite motif (AG)_n_, that was revealed by Tandem Repeats Finder^42^ analysis within the assembled genomes and the Illumina reads. About 37,000 loci of (AG)_n_ with an average length of 38 bp were detected in 8410, making up 1% of the assembly (the same proportion and characteristics were found for (AG)_n_ in the 7747 assembly). These dispersed simple repeat loci appeared in the *S. intermedia* genome with an average frequency of one per 3.7 kb.

### Characterization of rDNA loci

**Figure 6:**
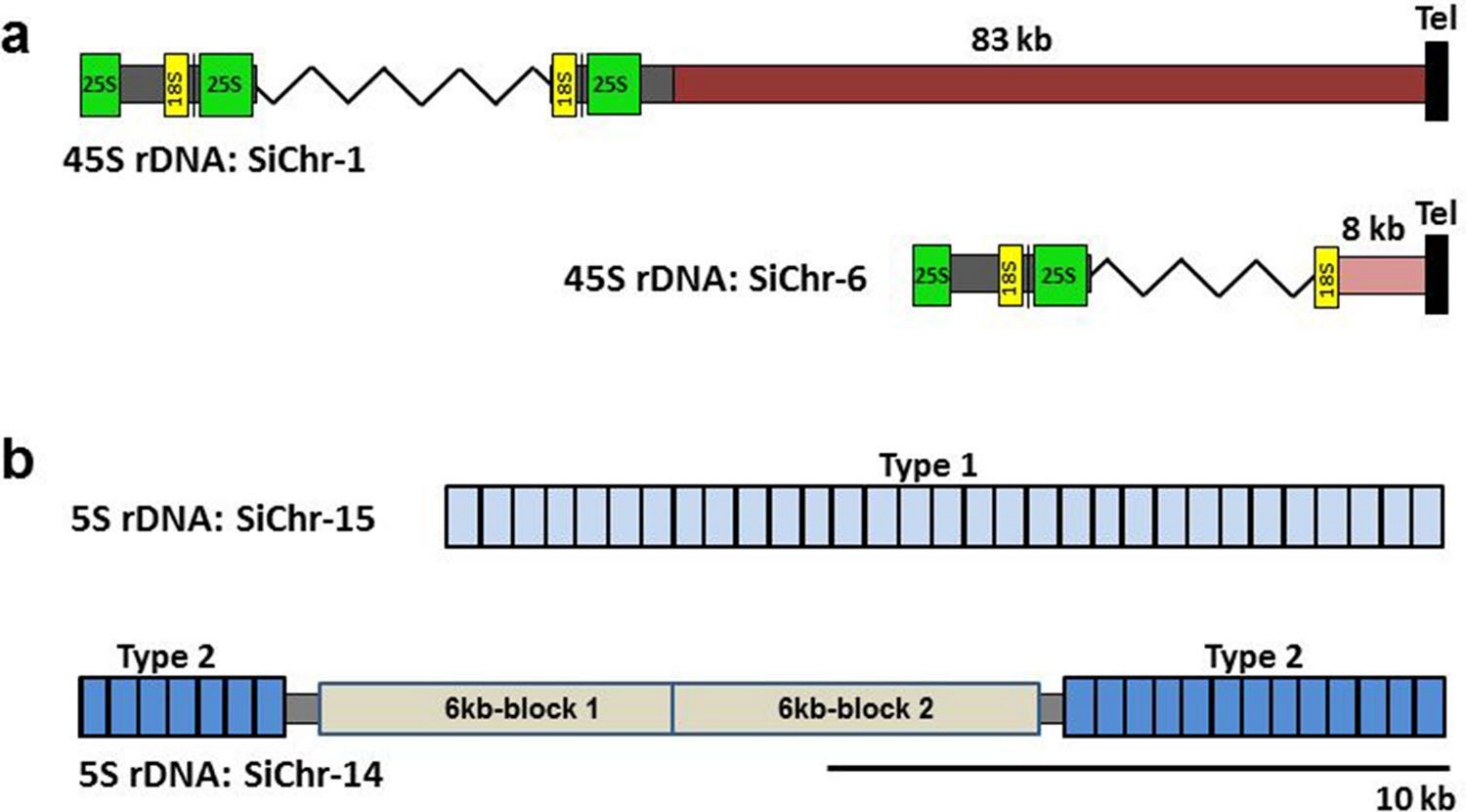
Schematic representation of rDNA loci in *S. intermedia* 8410. (**a**) Depiction of 45S rDNA loci at chromosomes ChrSi01 and ChrSi06; **Tel** = telomere. (**b**) Depiction of 5S rDNA loci at chromosome ChrSi15, and ChrSi14. The ChrSi15 locus contains 31 5S rDNA units with NTS of type-1; the ChrSi14 locus is composed of two clusters of 7 and 13 5S rDNA units of type-2 NTS, separated by a doubled 6 kb sequence of unknown function.

Chromosome analysis of *S. intermedia* by FISH using 5S and (18S + 26S) rDNA probes, detected one major locus of 5S rDNA on ChrSi15 (corresponding to ChrSp13) (Fig. S3a) and a major locus of 45S rDNA on ChrSi01 (corresponding to ChrSp01) (Fig. S3b). The chromosome-scale assembly and ON reads for Si8410 confirmed the presence of a 5S rDNA locus on ChrSi15, and revealed an additional locus on ChrSi14. The extra-long ON reads showed that the locus on ChrSi15 contains a cluster of thirty-one 5S rDNA repeats, whereas the locus on ChrSi14 is composed of seven and 13 5S rDNA repeats interrupted by two 6 kb long repeats of another sequence (Fig. 6b). The chromosome assembly, based on PacBio sequences, showed similar arrangement of 5S rDNA in Si7747 with slightly different copy numbers in each of the loci. Quantitative PCR estimation of 5S rDNA copy number in Si7747 and Si8410 genomes supports this data with 57 ± 10 copies of 5S rRNA genes for Si8410 and, 70 ± 20 copies for Si7747. Sequence alignments suggested that the 5SrDNA units of the two loci contain slightly different non-transcribed spacers (NTS). This was confirmed by sequencing of the 5S rDNA repeats, amplified from genomic DNA of clones 8410 and 7747. Analysis of individual 5S rDNA clones of Si8410 and Si7747 identified the 119 bp long 5S rRNA genes with 100% identity to the previously sequenced 5S rRNA gene of *S. polyrhiza*^43^, and two variants of the NTS for each ecotype (Fig. S4), with variant 8410-1 corresponding to 5S rDNA locus on chromosome ChrSi15 and the variant 8410-2 corresponding to the locus on chromosome ChrSi14 (Fig. 6a).

In agreement with the FISH data, the genome assembly revealed a 45S rDNA locus in a distal position on ChrSi01 for both clones, Si7747 and Si8410. Extra-long ON reads confirmed this locus for Si8410, about 83 kb upstream of one ChrSi01 chromosome telomere. Moreover, ON reads revealed an additional cluster of 45S rDNA repeats about 8 kb upstream the telomere on ChrSi06. Unlike the situation with 5S rDNA loci, there are no read-through sequences containing the whole 45S rDNA loci. Therefore, it is not possible to determine exactly how many of the 93 ± 25 45S rDNA copies estimated by qPCR for Si8410, are located in the minor locus.

## Discussion

### Chromosome rearrangements between the two Spirodela species

Previously, chromosome homeology and rearrangements between *S. polyrhiza* and *S. intermedia* were uncovered by comparative serial multicolor cross-hybridization to the *S. intermedia* clone 8410^29^. Here, we applied the same approach, using informative BACs out of the 96 ones anchored in the *S. polyrhiza* genome, and found the same chromosomal rearrangements in *S. intermedia* clone 7747 as reported for clone 8410^29^. Reiterative rounds of genome assembly and validation by FISH revealed a new linkage compared to the *S. polyrhiza* genome, in addition to the eight translocations detected previously^29^. This new linkage involves ChrSi16, which received small parts from one end of ChrSp20 (Fig. 2). A small piece, apparently transferred from one end of ChrSp13 to ChrSi02, turned out to be an error within the previous ON assembly for ChrSp13 of clone 9509^24^. Another small region, transferred from ChrSp14 to ChrSi04 was not studied by FISH, but it appeared in both assemblies. The *S. intermedia* chromosomes ChrSi03, 04, 06, 07, 09 and 11, previously found to be involved in evolutionary rearrangements, correspond in total to 10 *S. polyrhiza* chromosomes (Figs. 1, 2, 3 and^29^). Now, at least two more rearrangements (in addition to the previously postulated one inversion and eight translocations) have to be assumed if the *S. polyrhiza* karyotype is more similar to that of the ancestor. Alternatively, eight (instead of six) translocations (involving ChrSi03, 04, 06, 07, 08, 11, 12, 16, 18), and one fission (ChrSi09)^29^ were required if the *S. intermedia* karyotype is the more ancestral one. Additionally, we found a positional change of the smaller 5S rDNA locus to ChrSi14 (instead of ChrSi07 which harbors sequences adjacent to the minor 5S rDNA locus on ChrSp06).

The increased number of rearrangements refined our knowledge about karyotype evolution between *S. polyrhiza* and *S. intermedia* and made the corresponding genome assembly for both *S. intermedia* clones robust by independent confirmation. These results demonstrate the benefit of our novel approach, combining comparative cytogenomics and hybrid sequencing technologies, especially for species for which genetic maps are not available.

### Genome assembly for both S. intermedia clones

The assembly of PacBio reads for clone 7747 is more fragmented than the assembly of ON/Illumina reads for clone 8410. It displays a higher percentage of ambiguous bases (0.003%) and a lower coverage (Table 1). Several draft contigs representing contamination from plastids, mitochondria, and a virus (Human alpha herpesvirus 1 strain) were detected which show largely increased coverages of 2925-, 17,126- and 73-fold respectively. These overrepresented sequences caused a diminished overall coverage in both assemblies. Canu performs best with more than 50X coverage, while lower values might decrease the contiguity of an assembly^32^.

Comparing read length N50 of the two S. intermedia clones, the ON library of clone 8410 (16,322 kb) yielded a larger amount of long reads than clone 7747 with an N50 of 9879 kb. As a consequence, longer repeats may not be sequenced completely, leading to a more fragmented assembly^44^, as in case of clone 7747.

In summary, the PacBio read assembly of *S.intermedia* 7747 provides continuous pseudomolecules but these might comprise ambiguous and low coverage nucleotide stretches, and possibly missing sequences, as described for other plant species^45^. Despite the lower library quality for clone 7747, the non-hybrid PacBio assembly shows (as expected^46^) good results for the scaffold N50 (8.3 Mbp similar to 9.25 of the ON/Illumina assembly, Table 1), depicting the synergy of cytogenetics and long read sequencing technologies.

The distribution of functional eggNOG groups within the *S. intermedia* clones and *S. polyrhiza* is largely balanced, indicating a high similarity in overall functional gene content between both species which amounts with ~ 20,000 genes, a rather low number compared to most land plants. The BUSCO results reflect the increased fragmentation of the 7747 assembly by an increased number of fragmented and missing genes (~ 20% in total). With 3.0% of fragmented and 3.9% of missing candidate genes, the ON based assembly of clone 8410 exhibits a good representation of the expected gene content. It has to be noted, that the embryophyta set of orthologue sequences (mostly from land plants) might not be optimal for aquatic plants, because unnecessary genes might have been lost during evolutionary organ reduction and aquatic life style of duckweeds^21^.

### Repetitive elements

*S. intermedia* revealed a rather low proportion of detected repeats (~ 25%), in comparison with other genomes of similar size, such as that of *A. thaliana* (157 Mbp/1C; 32%^47^ ), or the even smaller genome of *Genlisea nigrocaulis* (86 Mbp/1C) with 15.9% of total repeats^48^. Similar repeat proportions were reported by Wang et al. (2014)^21^ for *S. polyrhiza*, however, later Michael et al. (2017)^25^ reported a higher proportion of mobile elements (25%) for that species. Possible explanations might be: (1) There is a difference in repeat content between *S. polyrhiza* and *S. intermedia;* this seems less likely, because of the close relationship and same genome size of both species. (2) The assembly by Michael et al.^25^ is more complete; this is probably not the case, because we found a lower content of mobile elements also in unassembled reads. (3) Their methods including structure-based detection revealed low copy elements (one or a few copies/C) which are not captured by our clustering and similarity-based searches. (4) Michael et al.^25^ described a ratio of soloLTRs to complete elements of 8 to 1. Because our tools did not determine soloLTRs, the repeat content for *S. intermedia* might be underestimated. A high proportion of soloLTRs suggests that the genome size of Spirodela decreased by deletion-biased DNA double-strand repair^49^ via the single-strand annealing pathway since its separation from the other duckweed lineages. Remarkable is the high abundance of SSR repeats, especially that of dispersed (AG)_n_ microsatellite arrays, which in rice contribute to regulate gene expression by binding transcription factors^50^. Such arrays were reported also for *S. polyrhiza*, where they “severely impeded elongation during sequence assembly”^21^.

The unusually low copy number of both 45S and 5S rDNA repeats revealed in the genome of *S. polyrhiza*^24,25^, inspired curiosity about the number and arrangement of those genes in related *S. intermedia*. The qPCR-based estimation showed ~ 93 copies of the 25S rRNA genes for both Si8410 and Si7747 genomes, a number very close to the estimate for *S. polyrhiza*^25^*.* Unlike *S. polyrhiza*, where the 45S rDNA was shown to locate in a single chromosome locus, our deep coverage ON sequencing of Si8410 revealed at least two 45S rDNA loci, located at ~ 83 kb upstream of the telomere at SiChr01, and at ~ 8 kb upstream of the telomere at SiChr06. The fact that FISH revealed only one signal on SiChr01, suggests that this location is the major 45S rDNA locus with the majority of the ~ 93 gene repeats. However, the exact 45S rDNA copy number distribution between the two loci remains unclear, because no read-through sequences are available. For the 5S rDNA, a range of generated ON read-through sequences, revealed two distinct loci, one containing a cluster of 31 repeats on SiChr15 which has been also visualized by FISH, and a second split locus on SiChr14 containing two clusters with 7 and 13 repeats, separated by ~ 13 kb long region of non-rDNA sequence (Fig. 6b). While the general arrangement of the 5S rRNA genes in *S. intermedia* resembles that of the *S. polyrhiza*, the total 5S rDNA copy number in *S. intermedia* is even lower, 51 vs 73 in *S. polyrhiza*^24^. This is the smallest number reported so far for any plant species. Usually, the copy number of 5S RNA genes in land plants varies from 2000 to 75,000^51^. Therefore, our findings for *S. polyrhiza* and *S. intermedia* proclaim that the small copy number of rDNA is a unique phenomenon of the genus Spirodela.

## Material and methods

### Plant material

*Spirodela intermedia* W. Koch (accessions 8410 from Panama City and 7747 from Lima, Peru) were obtained from Elias Landolt’s collection via Klaus Appenroth, University of Jena and Rutgers Duckweed Stock Cooperative (New Jersey, USA). The fronds were grown in liquid nutrient medium^52^ under 16 h white light of 100 µmol m^−2^ s^−1^ at 24 °C.

### Genomic DNA isolation

Genomic DNA of *S. intermedia* (clone 7747) was extracted from fresh fronds using the DNeasy Plant Mini Kit (Qiagen) for PacBio sequencing. High molecular weight DNA of *S. intermedia* (clone 8410) was isolated for Oxford Nanopore and Illumina sequencing as follows: the plants were kept three days in the darkness. After harvesting, 10 g of the fronds were used for DNA isolation from purified nuclei according to the protocol of Vondrak et al.^53^, with two minor modifications: the centrifugation of nuclei was performed at 650 × g instead of 200 × g due to their small size, and 2 × CTAB isolation buffer was supplemented with 2% PVP-360 (polyvinylpyrrolidone, avg. molecular weight 360,000).

### Genome sequencing

#### PacBio

After shearing of genomic DNA (*S. intermedia* clone 7747), a size-selected 20 kb library was sequenced on the Pacific Biosciences RS II platform (GATC Biotech, Konstanz, Germany) combining the P6-C4 polymerase-chemistry and 240 min of movie duration. Two rounds of sequencing resulted in 149 Gb of raw read data.

#### Oxford nanopore

The sequencing libraries were prepared from 3 μg of purified HMW DNA using a Ligation Sequencing Kit SQK-LSK109 (Oxford Nanopore Technologies) as described by Vondrak et al.^53^. Briefly, the DNA was treated with 2 μl of NEBNext FFPE DNA Repair Mix and 3 μl of NEBNext Ultra II End-prep enzyme mix in a 60 μl volume that also included 3.5 μl of FFPE and 3.5 μl of End-prep reaction buffers (New England Biolabs). The reaction was performed at 20 °C for 5 min and 65 °C for 5 min. Then, the DNA was purified using a 0.4 × volume of AMPure XP beads (Beckman Coulter). Because long DNA fragments caused clumping of the beads and were difficult to detach, the elution was performed with 3 mM TRIS–HCl (pH 8.5) and was extended up to 40 min. Subsequent steps including adapter ligation using NEBNext Quick T4 DNA Ligase and library preparation for sequencing were performed as recommended. The whole library was loaded onto FLO-MIN106 R9.4 flow cell and sequenced on MinION instrument until the number of active pores dropped below 40 (21–24 h). Basecalling of the raw reads was done using Albacore 2.3.3 (Oxford Nanopore Technologies).

#### Illumina

Illumina sequencing was performed by Admera Health, LLC (South Plainfield, NJ, USA) using a KAPA DNA Library kit (Roche) and resulted in 130 million paired-end reads (2 × 150 nt).

### Sequence assembly and pseudomolecule construction

After an initial filtering for potential bacterial contamination (blastn against microbial NCBI refseq database from Aug 2017) and minimum read length (500 nucleotides), PacBio reads were assembled using the Canu pipeline v. 1.5^32^ (options: ‘genomeSize = 160 m, correctedErrorRate = 0.105′) consisting of the following steps:

1. Trimming, error correction and contig construction: Reads were corrected and trimmed by comparing overlaps. A minimum length of 500 nucleotides and a maximum error rate of 10.5% was chosen for extending a contig. Only reads consisting of more than 1000 nucleotides in length were considered in this step. Afterwards, the corrected reads were trimmed to improve overall read quality by using overlap information to detect high confidence regions. Contigs of insufficient read coverage and/or containing ‘noisy’ sequence were categorized as ‘unsupported regions’ and divided at weak sequence positions into subcontigs with higher support.
  After further contig construction on the basis of overlaps, a consensus sequence was built by removing the remaining sequencing errors to raise the overall assembly quality.
2. *Scaffolding and gap filling*
  In a first round of scaffolding, the two genomes of the sister species *S. polyrhiza* (from clones 9505 and 7498)^23,25^ were used as references for Mauve Genome Aligner v20150522^33^ to order contigs. Scaffolding was performed by SSPACE-Longread v.1-1^34^ (default options). The resulting scaffold assembly was used for the super-scaffolding approach. For this aim, contigs were assigned to 18 putative pseudomolecules (corresponding to the 18 *S. intermedia* chromosomes) using the information of cross-FISH of 93 *S. polyrhiza* BACs on the chromosomes of *S. intermedia* clone 8410^29^. New cytogenetic probes using BACs from the genomic regions of interest were designed for FISH experiments to approve localization of the contigs within the pseudomolecules and to resolve mis-assemblies. Additionally, bacterial contamination was filtered as described previously.

The quality of both *S. intermedia* genome assemblies was assessed by the BUSCO program^36,37^ with the Embryophyta odb10 dataset comprising 1375 conserved genes.

The ONT/Illumina assembly for clone 8410 was performed using MaSuRCA^54^. In the first step, super-reads were assembled from 68 million Illumina 2 × 151 nt paired-end reads (128-fold coverage). Subsequently, 307,111 nanopore reads 10,000–425,377 nt in length (total length 9,963,752,003) and representing a 62-fold genome coverage, were used in scaffolding step. The resulting MaSuRCA assembly consisted of 386 scaffolds (N50 408,333 bp, total length 191,862,084 bp). Scaffolds were assigned/ordered into pseudomolecules using the 7747 as reference for a Mauve Genome alignment^33^. Additionally, all the formation of super-scaffolds was accompanied by manual curation steps based on FISH results.

### Gene prediction and functional annotation

Gene finding was carried out using Gene Model Mapper (GeMoMa)—a similarity-based gene prediction program^39^ (‘GeMoMa-1.6.1.jar CLI GeMoMaPipeline t = g = a = Extractor. p = true AnnotationFinalizer.r = SIMPLE AnnotationFinalizer.p = ’). Gene models were predicted by combining the predictions based on the genome data of the neighbor species *S. polyrhiza* and eight additional reference organisms (*S. polyrhiza* 7498 v3.1^23^, *Lemna* minor 5500^38^, *Arabidopsis thaliana* TAIR10, *Ananas comosus* (Phytozome internal code 321), *Brachypodium distachyon* (Phytozome internal code 314), *Nelumbo nucifera* 1.1 (GenBank assembly accession: GCF_000365185.1), *Panicum hallii* v3.1 (GenBank assembly accession: GCF_002211085.1), *Oryza sativa* IRGSP v1.0.38 (GenBank assembly accession: GCA_001433935.1), *Zostera marina* v2.1 (GenBank assembly accession: GCA_001185155.1)).

Noncoding RNAs were determined using RNAmmer v1.2^55^ (‘rnammer -S euk -m tsu,ssu,lsu -h –f’) and tRNAscan-SE-2.0^56^ (‘tRNAscan-SE -B -o -b –thread 10′). Predicted tRNAs were filtered for features overlapping with protein coding exons (‘bedtools intersect -b -a -wa -wb -f 0.8 -r’).

Functional annotations and GO terms were assigned using Interproscan 5 v 5.26–65.0^57^ (‘interproscan.sh -dp -input -seqtype p -f tsv,html,gff3 -applications TIGRFAM, PfamA, SMART, SUPERFAMILY –pathways –goterms’). COGs (clusters of orthologous groups) were computed using eggnog-mapper^58^ online tool (‘Taxonomic background: Viridiplantae, default options’) based on eggNOG 4.5 orthology data^59^.

### Comparative genomics and visualization

Best bi-directional hits (BBHs) were identified using BLASTN 2.2.31 + (‘-evalue 1e-5 -out outfmt 6′). Additional filtering for length and percent identity was applied afterwards (minimum length: 100 nt; minimum identity: 80%). Annotation of gene features and repetitive elements in *S. polyrhiza* 9505 was taken from Michael et al., 2017^25^. Both, length of selected repeat features and gene density were computed across a 0.5 Mbp window using the bedops suite^60^.

Feature annotation of each annotated repeats and genes as well as chromosome sizes have been converted to bed format using ‘gtf2bed’. From these files the 0.5 Mbp windows across each chromosome have been calculated with bedops –chop. Finally, the counts of gene and repeat features within each chromosome has been determined by bedmap –count.

Synteny was determined using minimap2 (‘minimap2 –x’)^35^ between *S. polyrhiza* and *S. intermedia*. 8410. Overlapping intervals have been merged by bedtools^61^ merge (default options). Links shorter than 10.000 have been excluded from the analysis.

To compare the two Spirodela genomes, synteny between pseudomolecules, gene and repeat distribution have been plotted using Circos tool v0.67-1^62^.

### Repeat annotation and analysis

Repeat analysis in unassembled Illumina reads from the clone 8410 was performed using similarity-based clustering implemented in the RepeatExplorer pipeline^40^. The pipeline was run with modified settings in order to increase its sensitivity towards divergent repeats. The similarity search step was done with BLASTN instead of mgblast and the similarity threshold was lowered from 90 to 80% identity. A total of 2.4 million of 150 nt paired-end reads were used as an input, and repeats were annotated in clusters representing at least 0.005% of the input reads.

Repeat annotation in the assembled pseudomolecules was performed using several alternative approaches. PROFREP and DANTE modules available at the public RepeatExplorer server (https://repeatexplorer-elixir.cerit-sc.cz/) were used to annotate repeats based on similarities to the reference database compiled from the repeat clustering analysis described above, and to the REXdb database of conserved protein domains of mobile elements^63^, respectively. Additionally, repetitive sequences in the assemblies were annotated using RepeatScout v. 1.0.5^41^. First, a library of repeats based on frequent k-mers was created using default parameters. Repeats with a frequency below 10 copies were then removed from the library. The resulting library was then used for annotating genome assemblies using RepeatMasker (https://www.repeatmasker.org).

Identification and quantification of microsatellites (simple sequence repeats) was done by means of Tandem Repeat Finder^42^ using the settings “trf input_file 2 5 7 80 10 25 25 -f” and parsing the output using TRAP^64^.

### Molecular characterization of rDNA

The estimation of 25S and 5S rRNA gene copies was carried out by qPCR, relating the rates of sample DNA amplification to the standard curve. The standard curve was constructed based on the amplification reads of dilution series of a reference plasmid, containing part of the *actin* gene (single copy in *Spirodela polyrhiza, 9509*), a whole PCR amplified 5S rDNA unit of *S. polyrhiza* and a part of the 25S gene amplified from genomic DNA of *S. polyrhiza*, 9509, using primers with internal restriction sites for XbaI and EcoRI. The rDNA copy number was determined in qPCR reactions prepared with the UltraSybr Mixture (CWBio, Taizhou, China), run on the CFX Connect Real-Time detection system (Bio-Rad, Hercules, USA). For quantification of the 25S rDNA we used the 5′-TCCCACTGTCCCTGTCTACT and 5′-CCCACTTATCCTACACCTCT primers, and for the 5S rDNA the set of primers was: 5′-GGGTGCGATCATACCAGCAC and 5′-GGGTGCAACACGAGGACTTC. The samples and tenfold dilution series of the reference plasmid were assayed in the same run. The quality of products was checked by thermal denaturation cycle. Only the experiments providing a single peak were considered. Three technical replicates were performed for each sample. The obtained data were analyzed using the program BIO-RAD CFX Manager 3.1 (Hercules, USA) and Microsoft Excel 2016 software.

For sequencing, the 5S rDNA genes were amplified from genomic DNA by PCR using 5S rRNA gene-specific primers DW-5S-F: CTTGGGCGAGAGTAGTACTAGG and DW-5S-R: CACGCTTAACTTCGGAGTTCTG, purified by gel electrophoresis and cloned into the vector pMD19 (Takara, Dalian, China). The obtained sequences were analyzed using the “Online Analysis Tools” package (https://molbiol-tools.ca).

### Mitotic chromosome preparation, probe preparation and FISH

Spreading of mitotic chromosomes was carried out according to Hoang^29^. 5S rDNA, 18S and 26S rDNA probes were generated from *S. polyrhiza* and from *S. intermedia* genomic DNA each by using designed primer pairs^16,21,65,66^ as described^24^. Ribosomal DNA, *A. thaliana* type telomere and *S. polyrhiza* BAC probes were labeled with Cy3-dUTP (GE Healthcare Life Science), Alexa Fluor 488-5-dUTP, Texas red-12-dUTP, biotin-dUTP or digoxigenin-dUTP (Life Technologies) and precipitated as described^29^.

Denaturation of mitotic chromosomes and probes, hybridization, post-hybridization washing and signal detection were carried out according to Lysak et al.^67^. Probe stripping and re-hybridization were done as described^29^.

### Microscopy and image processing

Fluorescence microscopy for signal detection followed Cao et al.^23^. The images were processed (brightness and contrast adjustment only), pseudo-colored and merged using Adobe Photoshop software ver.12 × 32 (Adobe Systems).

## Supplementary information

Supplementary Information.

## Data Availability

The genome assemblies of S. intermedia 7747 and 8410 have been deposited at the European Nucleotide Archive (ENA) under PRJEB35514 and PRJEB35634, respectively. Raw reads can be obtained from EBI ENA using accession numbers PRJEB33624 (PacBio, *S. intermedia* 7747), ERR3829756 (Illumina, *S. intermedia* 8410), and ERR3957957-ERR3957958 (Oxford Nanopore, *S. intermedia* 8410).
