# Supplementary material for: Chromosome-scale genome assembly for the duckweed Spirodela intermedia, integrating cytogenetic maps, PacBio and Oxford Nanopore libraries
Source: Sci Rep. 2020 Nov 5;10:19230. doi: 10.1038/s41598-020-75728-9 (PMC7645714; doi:10.1038/s41598-020-75728-9)
Supplement: Supplementary file 1 — Supplementary Information. [file 41598_2020_75728_MOESM1_ESM.pdf]

Chromosome-scale genome assembly for the duckweed *Spirodela intermedia*, integrating cytogenetic maps, PacBio and Oxford Nanopore libraries

Phuong TN Hoang[1,2,+], Anne Fiebig[1,+], Petr Novák[3], Jiří Macas[3], Hieu X Cao[1,4], Anton Stepanenko[5,6], Guimin Chen[5,6], Nikolai Borisjuk[5,6], Uwe Scholz[1], Ingo Schubert[1*]

[1] Leibniz Institute of Plant Genetics and Crop Plant Research (IPK), Gatersleben, D-06466 Stadt Seeland, Germany

[2] permanent address: Biology Faculty, Dalat University, District 8, Dalat City, Lamdong Province, Vietnam

[3] Biology Centre, Czech Academy of Sciences, Institute of Plant Molecular Biology, České Budějovice, CZ-37005 Czech Republic

[4] present address: Martin-Luther-University Halle-Wittenberg, Institute of Biology, D-06120, Germany

[5] Jiangsu Key Laboratory for Eco-Agricultural Biotechnology around Hongze Lake, School of Life Sciences, Huaiyin Normal University, Huai'an, 223300, China

[6] Jiangsu Collaborative Innovation Centre of Regional Modern Agriculture & Environmental Protection, Huaiyin Normal University, Huai'an, 223300, China

+These authors contributed equally to this work

* Corresponding author: schubert@ipk-gatersleben.de, Tel: +49 394825239

IS: ORCID ID 0000-0002-6300-2068; AF: ORCID 0000-0003-3159-3593; US: ORCID 0000-0001-6113-3518; HC: ORCID ID 0000-0003-1230-4127; PTNH: ORCID ID 0000-0002-7418-9091; AS: ORCID ID 0000-0003-2326-6613; NB: ORCID ID 0000-0001-5250-9771; JM: ORCID ID 0000-0003-0829-1570; PN: ORCID ID 0000-0002-5068-9681;

Table S1: Statistics of gene prediction and functional annotation

	S. intermedia clone 7747	*S. intermedia* clone 8410	*S. polyrhiza* clone 9509[a]
Total number of gene models	22.245	21,594	20,659
3’ truncated genes	38	8	149
5’ truncated genes	2,981	327	3,350
Internal stop codons	1	0	277
Number of coding transcripts	22,116	21,546	16,877
Proteins with functional annotation	15,754	15,558	12,352
Protein coding genes with GO terms	11,166	10,234	8,398
Protein coding genes with eggNOG	16,162	16,493	11,327
Proteins with Pfam annotation	15,908	15,165	8,302

a) data taken from Michael et al., (2017)[25]

Table S2: Number of genes associated with eggNOG functional categories

eggNOG category	*S. intermedia* 7747	*S. intermedia* 8410	*S. polyrhiza* 9509
**CELLULAR PROCESSES AND SIGNALING**			
[D] Cell cycle control, cell division,	268 (1.6%)	362 (2.0%)	199 (1.7%)
[M] Cell wall/membrane/envelope biogenesis	249 (1.5%)	224 (1.3%)	189 (1.6%)
[N] Cell motility	2 (0.0%)	2 (0.0%)	2 (0.0%)
[O] Post-translational modification, protein	1507 (9.0%)	1511 (8.6%)	1069 (9.1%)
[T] Signal transduction mechanisms	1523 (9.1%)	1523 (8.6%)	1065 (9.0%)
[U] Intracellular trafficking, secretion and	651 (3.9%)	600 (3.4%)	486 (4.1%)
[V] Defense mechanisms	133 (0.8%)	143 (0.8%)	82 (0.7%)
[W] Extracellular structures	56 (0.3%)	43 (0.2%)	41 (0.3%)
[Y] Nuclear structure	40 (0.2%)	37 (0.2%)	37 (0.3%)
[Z] Cytoskeleton	225 (1.3%)	235 (1.3%)	190 (1.6%)
**INFORMATION STORAGE AND PROCESSING**			
[A] RNA processing and modification	521 (3.1%)	693 (3.9%)	415 (3.5%)
[B] Chromatin structure and dynamics	156 (0.9%)	193 (1.1%)	116 (1.0%)
[J] Translation, ribosomal structure and	626 (3.7%)	723 (4.1%)	430 (3.6%)
[K] Transcription	1466 (8.7%)	1478 (8.4%)	1068 (9.1%)
[L] Replication, recombination and repair	535 (3.2%)	935 (5.3%)	275 (2.3%)
**METABOLISM**			
[C] Energy production and conversion	698 (4.2%)	540 (3.1%)	308 (2.6%)
[E] Amino acid transport and metabolism	435 (2.6%)	537 (3.0%)	306 (2.6%)
[F] Nucleotide transport and metabolism	134 (0.8%)	144 (0.8%)	114 (1.0%)
[G] Carbohydrate transport and metabolism	962 (5.7%)	993 (5.6%)	674 (5.7%)
[H] Coenzyme transport and metabolism	199 (1.2%)	202 (1.1%)	155 (1.3%)
[I] Lipid transport and metabolism	470 (2.8%)	511 (2.9%)	348 (2.9%)
[P] Inorganic ion transport and metabolism	431 (2.6%)	394 (2.2%)	338 (2.9%)
[Q] Secondary metabolites biosynthesis,	529 (3.1%)	556 (3.1%)	387 (3.3%)
**POORLY CHARACTERIZED**			
[S] Function unknown	4992 (29.7%)	5080 (28.8%)	3503 (29.7%)

Table S3: Average similarity, length and chromosomal assignment of whole BAC sequences from *S. polyrhiza* on *S. intermedia* assemblies from PacBio (clone 7747) and Hybrid ON/Illumina (clone 8410)

BAC	Sp9509 (ON)	Si7747 (PacBio)			Si8410 (Hybrid ON/Illumina)		
	ChrSp (=gi|)	ChrSi_7747	Ave. simil	length	ChrSi_8410	Ave. simil	length
**030D21**	**gi|1**	**Si01**	87,46	60112	**jcf159735_S01**	87,61	59666
**014L22**	**gi|1**	**Si01**	87,12	81806	**jcf159735_S01**	87,15	88220
**019A13**	**gi|1**	**Si01**	87,1	59976	**jcf159735_S01**	87,17	60103
**012O14**	**gi|1**	**Si01**	85,08	46416	**jcf159735_S01**	85,16	47916
**032E03.**	**gi|1**	**Si01**	86,56	45578	**jcf159735_S01**	86,17	48318
**003D17**	**gi|1**	**Si01**	85,91	50373	**jcf159735_S01**	85,75	51344
**004D21**	**gi|2**	**Si02**	86,27	53252	**jcf159572_S02**	86,3	58720
**005N05**	**gi|2**	**Si02**	86,53	74035	**jcf159572_S02**	97,39	67910
**037B02**	**gi|2**	**Si02**	87,35	68814	**jcf159572_S02**	87,64	69089
**003H12**	**gi|2**	**Si02**	84,25	30588	**jcf159572_S02**	84,72	28285
**021D24**	**gi|3**	**Si04**	85,37	52775	**jcf159718_S04**	85,57	52789
**002E10.**	**gi|3**	**Si04**	84,9	50015	**jcf159718_S04**	86,81	38546
**002K06**	**gi|3**	**Si03**	85,95	83901	**jcf159755_S03**	85,88	79771
**011P20**	**gi|3**	**Si03**	88,53	20882	**jcf159755_S03**	87,47	20336
**010J02**	**gi|3**	**Si03**	85,25	53304	**jcf159755_S03**	85	52948
**006F09**	**gi|4**	**Si05**	85,98	37528	**jcf159694_S05**	89,95	39275
**013D18**	**gi|4**	**Si05**	86,16	32980	**jcf159694_S05**	85,93	35398
**035L13**	**gi|4**	**Si05**	85,91	71580	**jcf159694_S05**	85,59	75905
**017E03.**	**gi|4**	**Si05**	85,49	49432	**jcf159694_S05**	85,82	46564
**030K22**	**gi|5**	**Si06**	83,8	35963	**jcf159561_S06_3**	84,25	35725
**027D17**	**gi|5**	**Si06**	85,39	27060	**jcf159561_S06_3**	85,17	28765
**009A15**	**gi|5**	**Si06**	85	53927	**jcf159561_S06_3**	85,67	45060
**009O20**	**gi|5**	**Si06**	85,77	54215	**jcf159561_S06_3**	86,46	59510
**023I01**	**gi|6**	**Si06**	86,17	45086	**jcf159561_S06_3**	86,13	45287
**033A10**	**gi|6**	**Si06**	83,85	116778	**jcf159633_S07**	83,35	5641
**002I04**	**gi|6**	**Si07**	85,72	39814	**jcf159633_S07**	86,06	38432
**026N01**	**gi|6**	**Si07**	85	42591	**jcf159633_S07**	85,31	42343
**035L19**	**gi|6**	**Si03**	86,39	9519	**jcf159755_S03**	86,41	9518
**014F03**	**gi|7**	**Si07**	83,47	26249	**jcf159633_S07**	84,43	24575
**035P21**	**gi|7**	**Si07**	85,98	42029	**jcf159633_S07**	85,94	45303
**004A04**	**gi|7**	**Si08**	84,86	42563	**jcf159638_S08**	85,42	41477
**030B11**	**gi|7**	**Si08**	86,29	37369	**jcf159638_S08**	86,93	39912

**013I04**	**gi|8**	**Si09**	86,95	54696	**jcf159258_S09**	86,3	61589
**006P24**	**gi|8**	**Si09**	84,6	22078	**jcf159258_S09**	84,57	21724
**032L08**	**gi|8**	**Si09**	86,25	67943	**jcf159258_S09**	86,17	73293
**034K03**	**gi|8**	**Si09**	86	76355	**jcf159258_S09**	86,22	77363
**004E01.**	**gi|8**	**Si09**	84,2	30708	**jcf159258_S09**	84,63	30343
**006L17**	**gi|8**	**Si09**	84,74	59271	**jcf159258_S09**	85,1	59976
**037H06**	**gi|9**	**Si10**	85,94	84892	**jcf159563_S10**	85,96	81218
**025O16**	**gi|9**	**Si10**	85,68	59148	**jcf159563_S10**	85,85	58340
**019G12**	**gi|9**	**Si10**	86,05	30432	**jcf159563_S10**	85,77	44605
**024C02**	**gi|9**	**Si10**	85,63	65769	**jcf159563_S10**	85,7	64611
**036C07**	**gi|9**	**Si10**	86,17	69591	**jcf159563_S10**	86,33	71742
**001D13**	**gi|9**	**Si10**	84,51	31843	**jcf159563_S10**	84,35	38379
**035P14**	**gi|10**	**Si11**	84,88	33727	**jcf159580_S11**	85,03	37048
**002B12**	**gi|10**	**Si11**	85,33	51252	**jcf159580_S11**	85,39	64004
**037K21**	**gi|10**	**Si11**	86,68	44661	**jcf159580_S11**	86,4	53612
**008G11**	**gi|10**	**Si11**	83,42	40388	**jcf159580_S11**	84,1	40591
**020G09**	**gi|10**	**Si12**	85,73	50729	**jcf159635_S12**	85,77	43981
**028B20**	**gi|10**	**Si12**	84,89	45472	**jcf159635_S12**	84,95	47152
**026G16**	**gi|10**	**Si12**	86,92	55062	**jcf159635_S12**	87,03	52791
**019I03**	**gi|11**	**Si13**	86,83	56535	**jcf159540_S13**	86,8	56717
**010P10**	**gi|11**	**Si13**	85,3	34147	**jcf159540_S13**	85,82	34902
**004G03**	**gi|11**	**Si13**	84,37	33178	**jcf159540_S13**	83,87	36537
**011L15**	**gi|11**	**Si13**	87,11	39402	**jcf159540_S13**	83,87	36537
**001C15**	**gi|11**	**Si13**	86,66	67380	**jcf159540_S13**	86,89	65754
**028L12**	**gi|12**	**Si14**	86,43	27269	**jcf159533_S14**	86,5525	27264
**035F17**	**gi|12**	**Si14**	86,53	74050	**jcf159533_S14**	86,36	82780
**030D24**	**gi|12**	**Si14**	84,89	26996	**jcf159533_S14**	88,6	29763
**010L16**	**gi|13**	**Si15**	86,95	57898	**jcf159593_S15**	86,07	69048
**008B06**	**gi|13**	**Si15**	83,88	19711	**jcf159593_S15**	84,19	20862
**031B15**	**gi|13**	**Si15**	84,76	35280	**jcf159593_S15**	85,19	36491
**020L20**	**gi|13**	**Si15**	86,1	60590	**jcf159593_S15**	86,1	63574
**003B08**	**gi|14**	**Si07**	84,94	68038	**jcf159633_S07**	86,1	69838
**036F14**	**gi|14**	**Si07**	86,23	57142	**jcf159633_S07**	86,51	53981
**040G15**	**gi|14**	**Si07**	85	71317	**jcf159633_S07**	85,2	72711
**037I18**	**gi|14**	**Si03**	85,5	22859	**jcf159755_S03**	85,34	25230
**006D12**	**gi|14**	**Si03**	84,21	42787	**jcf159755_S03**	84,54	29381
**011C23**	**gi|15**	**Si16**	80	19792	**jcf159680_S16**	79	25137

**021P03**	**gi|15**	**Si16**	87	28053	**jcf159680_S16**	86,22	33699
**020E12.**	**gi|15**	**Si16**	84,13	67518	**jcf159680_S16**	84,68	71876
**035L20**	**gi|15**	**Si16**	85	26169	**jcf159680_S16**	84,68	32086
**009J15**	**gi|16**	**Si11**	85,4	57130	**jcf159580_S11**	85,8	57445
**002L10**	**gi|16**	**Si11**	84,89	14497	**jcf159580_S11**	84,46	36142
**009O07**	**gi|17**	**Si04**	86,52	30662	**jcf159718_S04**	87,82	47243
**004N06**	**gi|17**	**Si04**	82,02	177344	**jcf159718_S04**	84,63	116454
**009L02**	**gi|17**	**Si04**	85,86	59364	**jcf159718_S04**	85,92	52240
**037B13**	**gi|18**	**Si09**	86,57	39229	**jcf159258_S09**	87,1	36333
**029K19**	**gi|18**	**Si09**	86,27	37344	**jcf159258_S09**	86,1	41667
**015L03**	**gi|19**	**Si17**	80,5	23281	**jcf159710_S17**	80,3	43506
**015J20**	**gi|19**	**Si17**	83,1	74308	**jcf159710_S17**	83,2	46935
**037H19**	**gi|19**	**Si17**	85,4	47128	**jcf159710_S17**	85,29	55129
**008A12**	**gi|19**	**Si17**	88,35	309	**jcf159710_S17**	88,39	310
**006C19**	**gi|20**	**Si18**	86,5	67472	**jcf159617_S18**	86,1	67065
**007A03**	**gi|20**	**Si18**	87,22	94416	**jcf159617_S18**	86,1	95006
**013O04**	**gi|20**	**Si16**	80,4	166821	**jcf159680_S16**	80,3	76462

Table S4: BLAST data of whole BAC sequences (029A10, 028I16 and 037J09) against assemblies for *S. polyrhiza* clones 7498 (Cao et al., 2016) and 9509 (Michael et al., 2017)

BAC	Sp7498 assembly							Sp9509 assembly						
	sseqid	pident	length	qstart	qend	sstart	send	sseqid	pident	length	qstart	qend	sstart	send
		99.53	22983	68796	91756	5822175	5799227		99.62	22173	118016	140148	5754022	5731861
		99.73	19450	91701	111136	5798870	5779431		99.67	7259	110726	117974	5761320	5754063
		99.41	16845	1	16811	5891712	5874872		99.81	8457	102185	110637	5769858	5761406
		99.44	15999	47861	63838	5843071	5827104		99.71	25957	76217	102154	5795842	5769889
		99.72	13817	111090	124885	5779242	5765428		99.66	8635	67463	76079	5804617	5795983
		99.44	13126	23910	37014	5867460	5854348		98.44	2812	64625	67430	5807531	5804728
		99.49	6476	41412	47887	5849805	5843358		99.48	7465	57275	64735	5814834	5807380
029A10	pseudo1	99.57	5640	128685	134314	5760759	5755130	CP019095.1	99.74	5039	52166	57202	5819952	5814919
		99.69	4789	16742	21528	5874847	5870060		99.76	1691	50392	52081	5821718	5820028
		99.16	4414	36977	41378	5854226	5849828		99.50	8912	41410	50312	5830711	5821829
		98.61	3964	64729	68670	5826171	5822215		99.82	2811	38560	41370	5833540	5830732
		99.63	2731	137420	140148	5751910	5749185		98.21	1728	35258	36971	5836919	5835201
		99.63	2424	21531	23951	5869905	5867484		99.74	12742	22467	35207	5849697	5836970
		99.61	2075	126653	128723	5763620	5761548		99.85	21478	1	21476	5872187	5850713
		99.10	1991	135482	137470	5754028	5752050							
		99.59	24197	19758	43939	5446478	5422300		99.61	46314	73255	119503	5284597	5238337
		99.35	12352	44831	57169	5420842	5408501		99.86	2933	84400	87331	5368531	5365600
		98.75	11240	72780	83999	5395318	5384128		98.85	6181	77798	83969	5375172	5369018
028I16	pseudo1	99.44	6081	13707	19784	5452735	5446667	CP019095.1	99.73	3745	73028	76766	5379992	5376248
		99.77	5730	3005	8729	5463838	5458112		99.42	5312	66925	72213	5386154	5380843
		99.64	5582	66903	72480	5400918	5395339		99.79	1881	64852	66732	5388291	5386412

		99.45	4361	9352	13707	5457181	5452830		99.09	4052	60074	64100	5396082	5392035
		99.77	3031	1	3031	5467227	5464200		99.38	9140	50541	59676	5405666	5396538
		99.28	2934	84399	87331	5383451	5380533		99.68	5554	44935	50483	5411269	5405722
		99.36	2661	57169	59822	5408303	5405649		99.69	5829	38709	44529	5417618	5411795
		96.94	687	8716	9402	5458170	5457498		99.75	13444	25037	38476	5431348	5417914
									99.79	9377	15604	24979	5440783	5431409
									99.04	1143	14399	15539	5441999	5440864
									99.44	5008	8716	13711	5447729	5442727
									99.68	8734	1	8729	5456401	5447671
		99.68	22753	107896	130634	5260327	5237617		99.82	5636	132201	137835	5222680	5217046
		99.61	18693	28622	47292	5346162	5327484		99.74	10705	120518	131220	5234356	5223666
		99.57	11934	8337	20263	5364343	5352425		97.71	2268	69628	71877	5288109	5285850
		99.32	10980	89724	100672	5278776	5267803		99.83	10044	59797	69834	5297991	5287948
		99.66	7641	62154	69788	5312112	5304473		98.29	2401	55331	57725	5302503	5300122
		99.46	7222	130621	137835	5237591	5230376	CP019095.1	99.38	7555	47340	54894	5310532	5303021
		99.63	6147	20223	26359	5352325	5346183		99.36	4694	42535	47225	5315344	5310653
		99.69	6052	79488	85532	5288615	5282568		99.19	3929	38822	42743	5319251	5315342
037J09	pseudo1	99.41	5428	1	5414	5373174	5367752		99.61	9682	28647	38324	5329400	5319727
		99.86	4323	103574	107896	5264870	5260548		99.69	7414	21179	28590	5336957	5329556
		99.37	3956	48408	52361	5326259	5322325		99.66	13713	7456	21145	5350686	5336985
		99.26	3537	85703	89235	5282315	5278780		99.36	7445	1	7431	5358179	5350747
		99.57	3058	74494	77544	5293937	5290881							
		99.89	2620	100651	103270	5267561	5264943							
		98.96	2681	5705	8369	5367163	5364484							
		99.64	2523	52310	54831	5321873	5319352		sseqid	Subject Seq-id (ID of the database hit)				
		98.84	2246	55326	57559	5318933	5316696		pident	Percentage of identical matches				

		99.53	2112	60048	62159	5314325	5312214		length	Alignment length					
		97.68	2203	69676	71853	5304636	5302439		qstart	Start of alignment in query					
		98.07	1662	58418	60068	5315977	5314333		qend	End of alignment in query					
		99.11	447	47966	48410	5326679	5326233		sstart	Start of alignment in subject (database hit)					
		96.77	155	74343	74497	5294056	5293904		send	End of alignment in subject (database hit)					
		98.18	55	89444	89498	5968585	5968531								
		100.00	50	86637	86686	5281330	5281281								
		100.00	50	86686	86735	5281379	5281330								

Fig. S1: DAPI-stained metaphase chromosome complements of *S. intermedia* clones 7747 and 8410 both showing 36 chromosomes.

Scale bars: 5µm

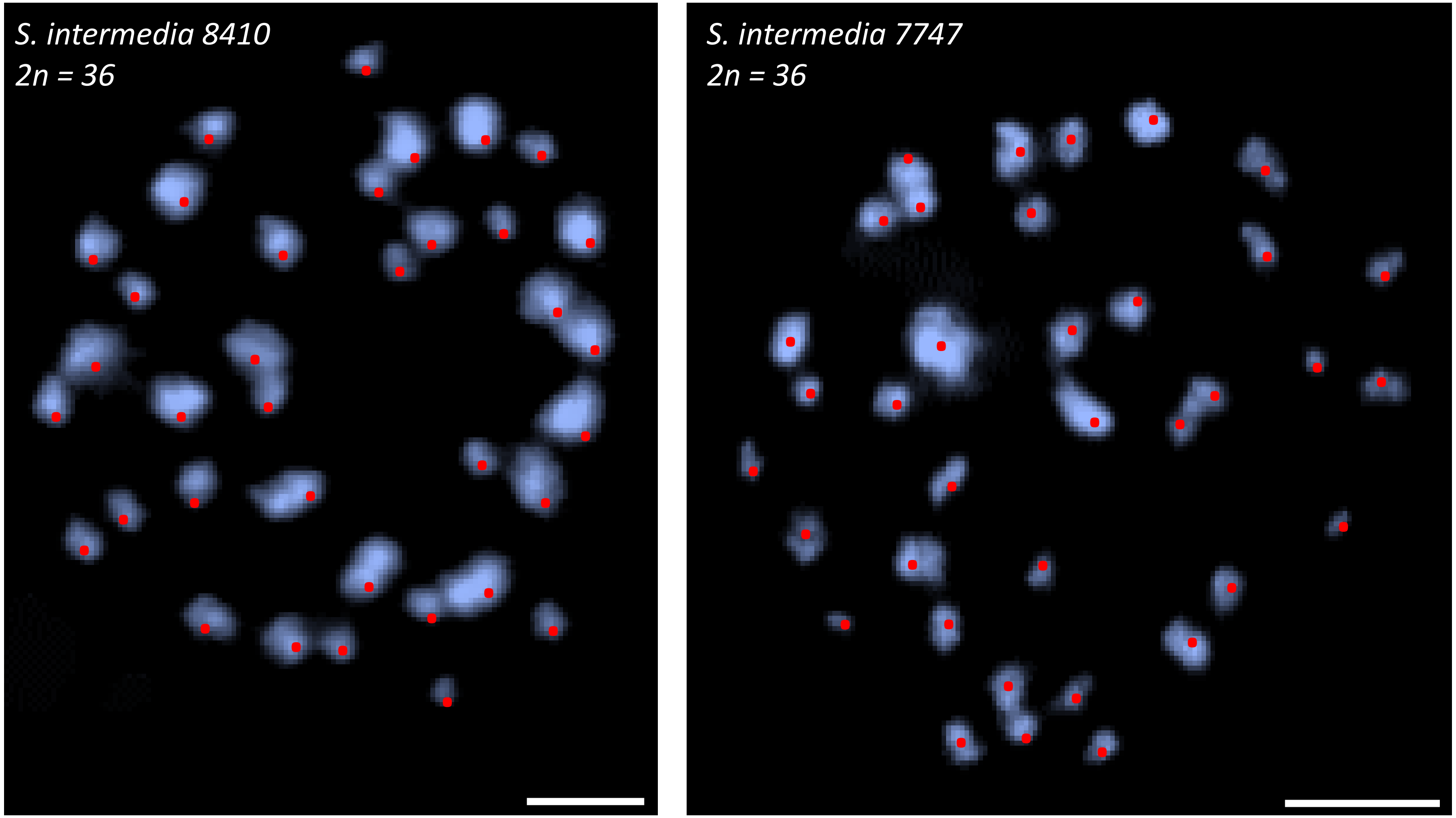

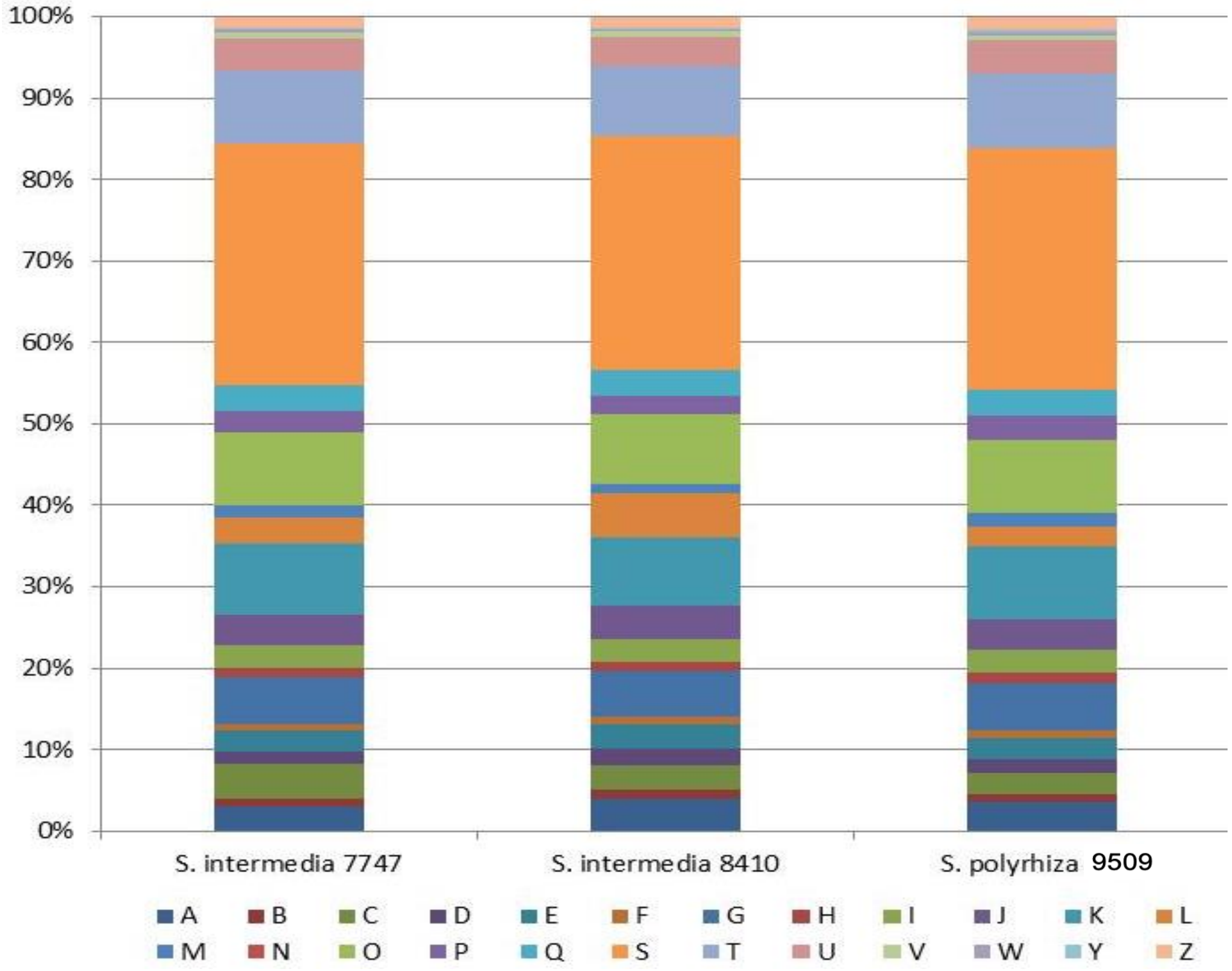

A	RNA processing and modification	M	Cell wall/membrane/ envelop biogenesis
B	Chromatin Structure and dynamics	N	Cell motility
C	Energy production and conversion	O	Post-translational modification, protein turnover, chaperone functions
D	Cell cycle control and mitosis	P	Inorganic ion transport and metabolism
E	Amino Acid metabolis and transport	Q	Secondary Structure
F	Nucleotide metabolism and transport	S	Function Unknown
G	Carbohydrate metabolism and transport	T	Signal Transduction
H	Coenzyme metabolis	U	Intracellular trafficing and secretion
I	Lipid metabolism	V	Defense mechanisms
J	Tranlsation	W	Extracellular structures
K	Transcription	Y	Nuclear structure
L	Replication and repair	Z	Cytoskeleton

Fig. S2: Percental distribution of genes associated with eggNOG functional categories within three *Spirodela* genomes.

Fig. S3: Conserved location of 5S and 45S rDNA on S. intermedia clones 8410 and 7747. (a) 5S rDNA is located on ChrSi 15 (corresponding to ChrSp 13) (b) 45S rDNA is located on ChrSi 01 (corresponding to ChrSp 01); Scale bars = 5µm

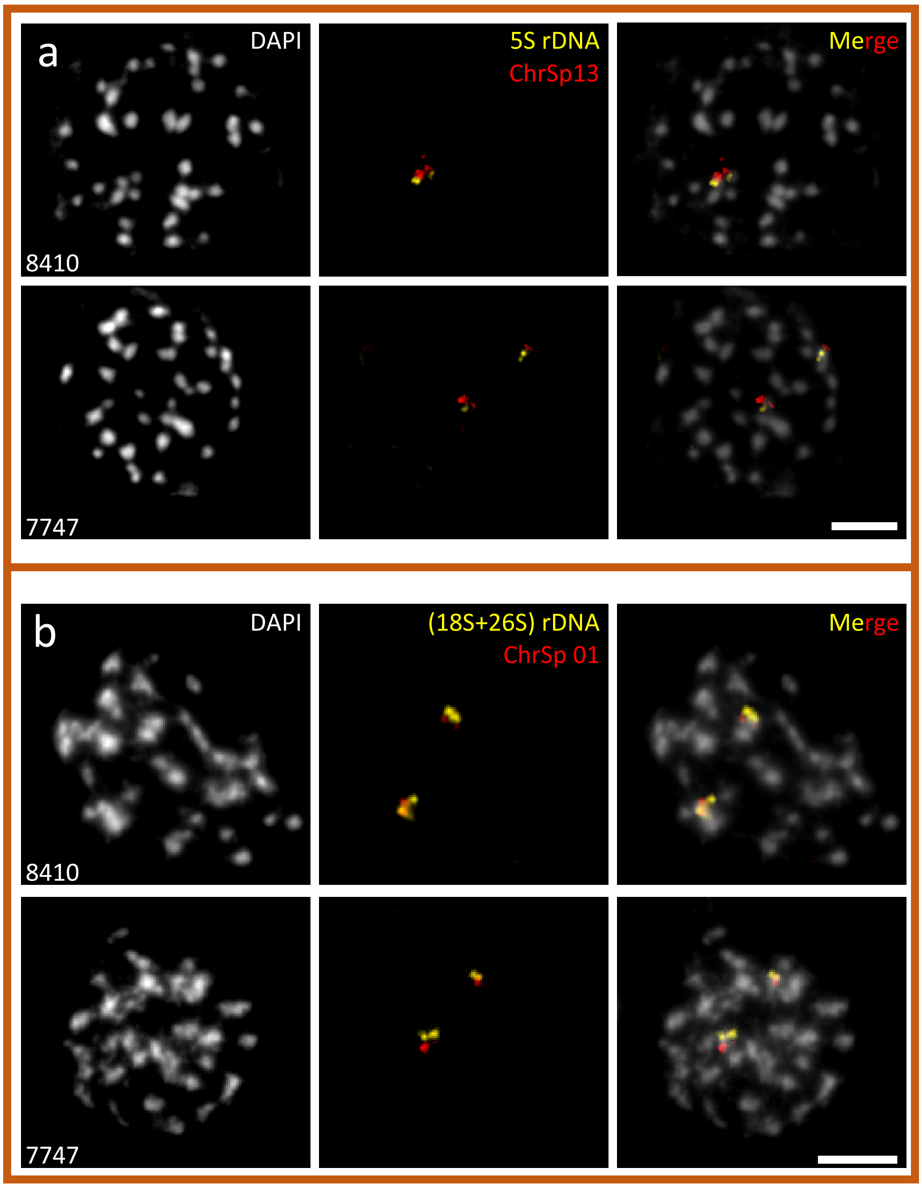

Fig. S4: Sequence alignment of two 5S rDNA units of *S. intermedia* clones 7747 and 8410. The 5S rRNA gene (in grey) is followed by the of non-transcribed spacer.

```
7747-1    gggtgcgatcataccagcactaatgcaccggatcccatcagaactccgaagttaagcgtg
8410-1    gggtgcgatcataccagcactaatgcaccggatcccatcagaactccgaagttaagcgtg
7747-2    gggtgcgatcataccagcactaatgcaccggatcccatcagaactccgaagttaagcgtg
8410-2    gggtgcgatcataccagcactaatgcaccggatcccatcagaactccgaagttaagcgtg
          ************************************************************

7747-1    cttgggcgagagtagtactaggatgggtgacctcctgggaagtcctcgtgttgcaccccc
8410-1    cttgggcgagagtagtactaggatgggtgacctcctgggaagtcctcgtgttgcaccccc
7747-2    cttgggcgagagtagtactaggatgggtgacctcctgggaagtcctcgtgttgcaccccc
8410-2    cttgggcgagagtagtactaggatgggtgacctcctgggaagtcctcgtgttgcaccccc
          ************************************************************

7747-1    ttccccctttttgcttgttgc---ttgttgctgccgcggaagtcgccgacagaccaaccc
8410-1    ttccccctttttgcttgttgc---ttgttgctgccgcggaagtcgccgacagaccaaccc
7747-2    ttccccccttttgctcgttgcttgttgttgctgccgctgaagtcgccgaccgac------
8410-2    ttccctcctttttgctcgttgcttgttgttgctgccgctgaagtcgccgaccgac------
          *****.*.*******.*****   ************* ************ ***     

7747-1    ccctatcctactatcctactacccctgtgagcgaggcgacttcctcgtccgctcggctct
8410-1    ccctatcctactatcctactacccctgtgagcgaggcgacttcttcgtccgctcggctct
7747-2    ------cccccctaccttattacccttgtgagcgaggcgacttcttcgtgcggtcggctct
8410-2    ------cccccctaccttattacccttgtgagcgaggcgacttcttcgtgcggtcggctct
                **. ***.*.**.******.*****************.**** ** ********

7747-1    ctacagcgcgttgtcgctacgaagctaaggacaagcgcaagctaactaactttcgggagt
8410-1    ctacagcgcgttgtcgctacgaagctaaggacaagcgcaagctaactaactttcgggagt
7747-2    ctacagcgcgttgtcgctacgaagccaaggacaagtgcaagctaactaactttcgggagt
8410-2    ctacagcgcgttgtcgctacgaagccaaggacaagtgcaagctaactaactttcgggagt
          *************************.*********.************************

7747-1    gtttcgggcacgtttgggcgcttaggaagggactcggaagacgggggccgcgcgttatgg
8410-1    gtttcgggcacgtttgggcgcttaggaagggactcggaagacgggggccgcgcgttatgg
7747-2    gtttcgggcacgtttgggcgcttaggaaggg-ctcggaagacgggggccgcgcgttatgg
8410-2    gtttcgggcacgtttgggcgcttaggaaggg-ctcggaagacgggggccgcgcgttatgg
          ****************************** *****************************

7747-1    gtgcggtgcgacctcccccagcggcgagagccatctcgcatctgcgctgctcggaggggc
8410-1    gtgcggtgcgacctcccccagcggcgagagccatctcgcatctgcgctgctcggaggggc
7747-2    gtgcggtgcgacctcccccagcggcgagagccatctcgcatctgcgctgcttgga-gggc
8410-2    gtgcggtgcgacctcccccagcggcgagagccatctcgcatctgcgctgctcggaggggc
          ***************************************************.*** ****

7747-1    gtcagcctccctcctcgtgggtcgatcgtctcacgggcccggcccggcccggcttggcga
8410-1    gtcagcctccctcctcgtgggtcgatcgtctcacgggcccggcccggcccggcttggcga
7747-2    gtcagcctccctcctcacgggtcgatcgtctcacgggcccggcccggcttggt-------
8410-2    gtcagcctccctcctcgtgggtcgatcgtctcacgggcccggcccggcccggcttggcga
          ****************..******************************..**.       

7747-1    tggaggtggagggagagagcggggggagagaatgagaccggatgcggcggatgtgacatc
8410-1    tggaggtggagggagagagcggggggagagaatgagaccggatgcggcggatgtgacatc
7747-2    ----gatggagggagagagcggggggagagaatgagatcggatgcggcggatgtgacatc
8410-2    tggaggtggagggagagagcggggggagagaatgagaccggatgcggcggatgtgacatc
              *.*******************************.**********************
```
